# A plasmonic nanorod that walks on DNA origami

**DOI:** 10.1038/ncomms9102

**Published:** 2015-08-25

**Authors:** Chao Zhou, Xiaoyang Duan, Na Liu

**Affiliations:** 1 Max Planck Institute for Intelligent Systems, Heisenbergstrasse 3, D-70569 Stuttgart, Germany

## Abstract

In nano-optics, a formidable challenge remains in precise transport of a single optical nano-object along a programmed and routed path toward a predefined destination. Molecular motors in living cells that can walk directionally along microtubules have been the inspiration for realizing artificial molecular walkers. Here we demonstrate an active plasmonic system, in which a plasmonic nanorod can execute directional, progressive and reverse nanoscale walking on two or three-dimensional DNA origami. Such a walker comprises an anisotropic gold nanorod as its ‘body' and discrete DNA strands as its ‘feet'. Specifically, our walker carries optical information and can *in situ* optically report its own walking directions and consecutive steps at nanometer accuracy, through dynamic coupling to a plasmonic stator immobilized along its walking track. Our concept will enable a variety of smart nanophotonic platforms for studying dynamic light–matter interaction, which requires controlled motion at the nanoscale well below the optical diffraction limit.

## Abstract (web summary)

The controlled movement of optically active components represents a challenge for nanooptical applications. Here the authors demonstrate a gold nanorod walker that performs directional walking along a DNA origami track and where the individual nanoscale steps can be monitored by optical spectroscopy in real time.

## Graphical abstract

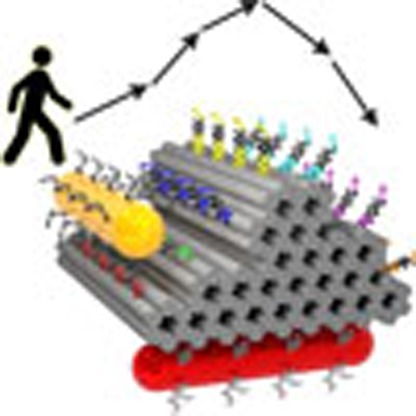

A gold nanoparticle that can walk along a prescriptive track is an active plasmonic system, which mimics the directional movement of naturally occurring molecular motors. Such a walker works not only as a walking element to carry out mechanical motion but also as an optical reporter, which can deliver its own translocation information through optical spectroscopy in real time. This may leverage the scope of synthetic molecular machinery^1^,^2^,^3^,^4^,^5^,^6^,^7^,^8^,^9^,^10^,^11^,^12^,^13^. Also, it circumvents the complexity of conventional walker characterization techniques as well as allows for noninvasive and stable characterizations over long periods of time.

In this study, we demonstrate an active plasmonic system, in which a gold nanorod can perform stepwise walking directionally and progressively on DNA origami. The nanoscale steps can be *in situ* monitored by optical spectroscopy. The key idea is to create a plasmonically coupled system, in which a walker and a stator, that is, an immobilized plasmonic element (or elements) on a walking track constitute a conformationally sensitive geometry^14^. When the walker carries out stepwise movements, it triggers a series of conformational changes of the system as well as activates subsequent near-field interaction changes with the stator, thus giving rise to immediate spectral response changes that can be read out optically. As a result, locomotion on the order of several nanometres, which is far below the optical resolution limit, can be optically discriminated in real time.

## Results

### Structural design of the walker system

**Figure 1:**
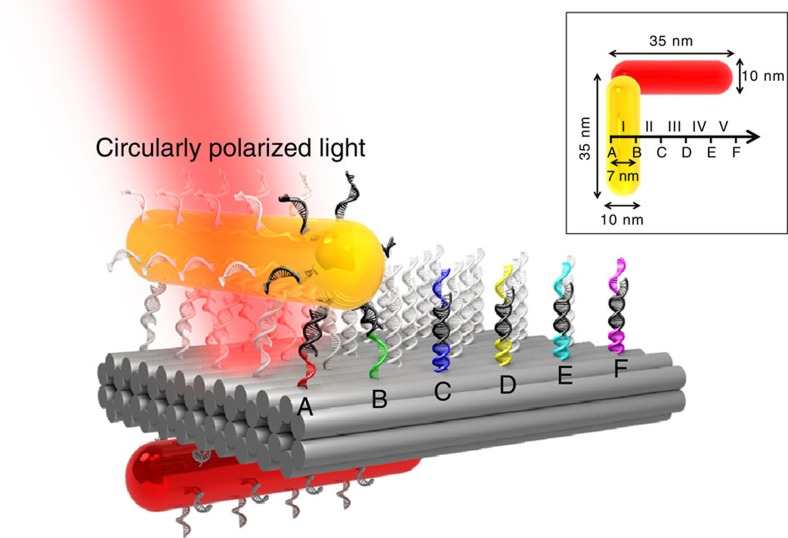
Schematic of the plasmonic walker. Two gold nanorods (AuNRs) are assembled perpendicularly to one another on a double-layer DNA origami template, forming a left-handed configuration at station I. The yellow AuNR on the top surface represents the ‘walker' and the red AuNR on the bottom surface represents the ‘stator'. The walking track comprises six rows of footholds (A–F) extended from the origami surface to define five walking stations (I–V). The distance between the neighbouring stations is 7 nm, which also corresponds to the step size. In each row, there are five binding sites with identical footholds. Only the footholds in the front line are coloured to highlight the different strand segments. The walker AuNR is fully functionalized with foot strands. To enable robust binding, the walker steps on two rows of neighbouring footholds at each station. The red beam indicates the incident circularly polarized light.

As shown in Fig. 1, a walker, gold nanorod (AuNR) in yellow and a stator (AuNR in red) are organized in a chiral geometry. Specifically, the walker and the stator are placed on two opposite surfaces of a two-dimensional (2D) rectangular DNA origami platform, forming a 90° cross configuration. A chiral geometry is chosen in that circular dichroism (CD), that is, differential absorption of left- and right-handed circularly polarized light, of three-dimensional (3D) chiral structures are markedly sensitive on their conformational changes^15^,^16^,^17^,^18^. DNA is used for robust self-assembly of anisotropic plasmoinc nanostructures^19^. The stator is immobilized on one surface through the capture strands on the origami, whereas on the other surface the walker can execute stepwise movements by programmably attaching (detaching) its feet on (from) the track through hybridization (de-hybridization) with the footholds (coded A–F in Fig. 1). In particular, double-layer DNA origami is utilized to achieve a rigid and robust track. The DNA origami (58 × 42 × 7 nm) was prepared by folding a long single-stranded DNA scaffold with staple strands and specific capture strands, following a self-assembly process^20^,^21^.

In contrast to previous tiny DNA walkers^1^,^2^,^3^,^4^,^5^,^6^,^7^,^8^,^9^,^10^,^11^, our walker comprises an anisotropic AuNR, which is as large as 35 × 10 nm. On one hand, a large metal nanoparticle is essential for plasmonic probing, as it yields distinct and pronounced optical response. On the other hand, the anisotropic nature of the AuNR also brings about substantial challenges to implement directional and progressive walking.

### Directional and progressive walking

To impose directional walking, the feet of the walker and the footholds on the track are specifically designed. The walker AuNR is fully covered with identical foot strands, which contain a nine nucleotide segment for hybridization and four thymine bases as spacer. Along the track, six parallel rows of footholds A–F are utilized to establish five walking stations I–V, which are evenly separated by 7 nm. This also defines the step size of the walker. At each station, the walker's feet step on two rows of the footholds to accommodate its transverse dimension as well as to ensure stable binding. In each row, five binding sites with identical footholds are extended from the origami. Each foothold consists of two parts: a binding segment (nine nucleotides, black) for hybridization with a foot strand of the walker as well as a toehold segment (eight nucleotides, coloured), which is differently sequenced in different foothold rows for achieving programmable reactions.

**Figure 2:**
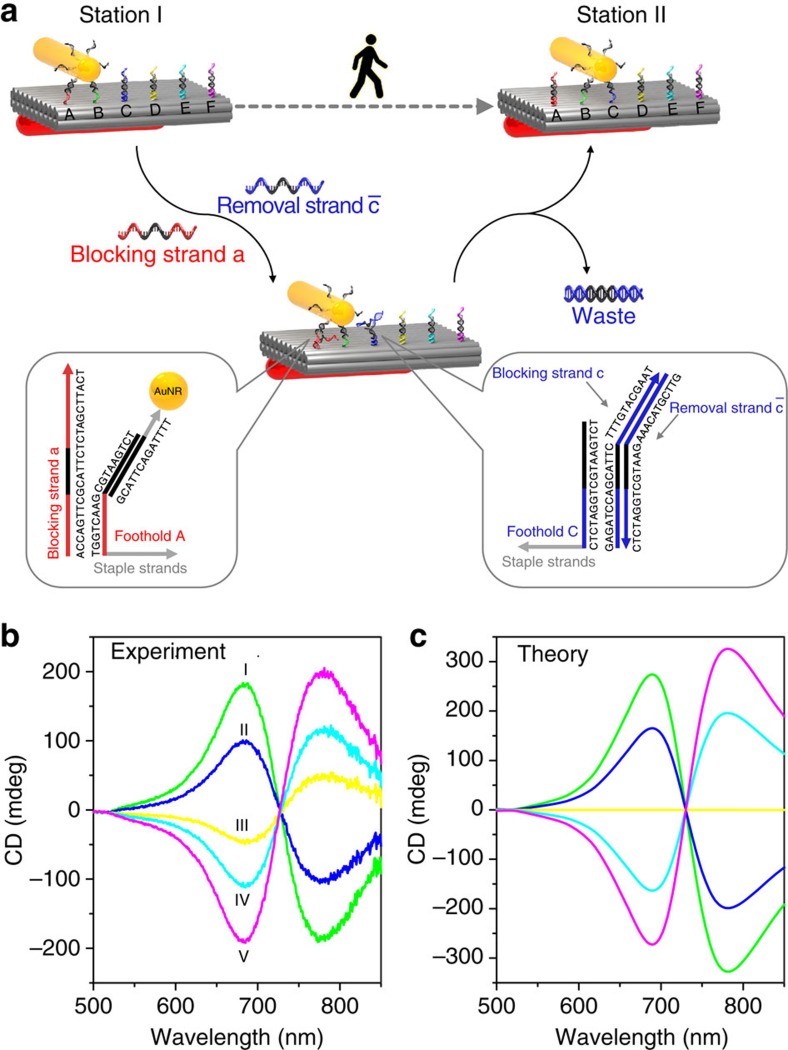
Walking mechanism, measured and simulated CD spectra at each station. (**a**) Walking mechanism. Initially, the walker resides at station I. On addition of blocking strand *a* and removal strand 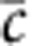, two toehold-mediated strand-displacement reactions occur simultaneously. Blocking strand *a* triggers the dissociation of the walker's feet from footholds A. Row A is then site-blocked. Meanwhile, removal strand 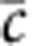 releases blocking strand *c* from footholds C. Row C is therefore site activated to bind the feet of the walker. Subsequently, the walker carries out one step forward, reaching station II. For simplicity, only the front line of the associated strands is shown. (**b**) Measured CD spectra at different stations. (**c**) Simulated CD spectra at different stations. The right-handed preference at station III was not included in the calculation.

Figure 2a schematically describes the walking principle. Initially, the walker resides at station I (start site), stepping on rows A and B through DNA hybridization. This was implemented with the assembly of the stator on the origami during the same annealing process. Foothold rows C–F are deactivated by respective blocking strands. At station I, the walker and the stator form a left-handed configuration. Due to close proximity, the two AuNRs can be strongly coupled. This generates a theme of handedness when interacting with left and right circularly polarized light, giving rise to CD^17^,^22^,^23^,^24^. To correlate discrete walking steps and their associated optical response, CD spectra at different walking stations were measured using a Jasco-815 CD spectrometer. All the measurements were carried out at room temperature and pH 8.0. The CD spectrum at station I is presented by a green curve in Fig. 2b, showing a characteristic peak-to-dip line shape centred around 740 nm. The measured CD intensity is as large as 200 mdeg at a sample concentration of ∼0.67 nM. Such strong and distinct spectral response enables highly sensitive spectroscopy, which is the basis to optically monitoring structural dynamics.

The stepwise walking is powered by DNA hybridization and activated on addition of respective blocking and removal strands. The blocking and removal strands for footholds A–F are labelled as *a*–*f* and 
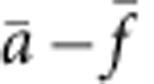
, respectively. Each blocking strand consists of three parts: the upper (11 nucleotides coloured), the middle (6 nucleotides, black) and the bottom segments (8 nucleotides, coloured). The removal strands are fully complementary to their corresponding blocking strands. First, blocking strand *a* and removal strand 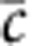 are added simultaneously. Dissociation of the walker's feet from row A is initiated by blocking strand *a* through a strand-displacement reaction mediated by the toehold segments on foothold A. Row A is then site-blocked. This eliminates the back stepping of the walker, thus imposing directionality. It is crucial to underline that blocking strand *a* is specifically designed so that the toehold segment of foothold A is fully hybridized with the bottom segment of blocking strand a, whereas the binding segment of foothold A is only partially hybridized with the middle segment of blocking strand *a*, leaving three nucleotides of the foothold unpaired. This ensures the specificity of the blocking strands for different foothold rows and more importantly avoids undesirable hybridization between the removal strands and the walker's foot strands.

Meanwhile, blocking strand *c* is dissociated from row C by removal strand 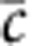 through branch migration, triggered by the upper segments of blocking strand *c* as toeholds. Row C is then activated. The walker's feet search for an accessible neighbouring site and subsequently bind to foothold C. As a result, the walker executes one step forward and reaches station II by stepping on rows B and C as shown in Fig. 2a. It is worth mentioning that during the process of detaching from row A and attaching to row C, one set of the walker's feet stay bound to row B, preventing the walker from being off the track. In a more descriptive picture, the walker imposes directional walking by alternatively advancing its feet in a ‘rolling' manner (For detailed description of the walking mechanism, see Supplementary Fig. 1 and Supplementary Note 1). At station II, the walker and the stator form a less asymmetric configuration compared with the case at station I. This leads to an immediate CD decrease as presented by the blue curve in Fig. 2b, indicating forward motion of the walker.

To impose progressive walking, blocking strand *b* and removal strand 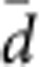 are added subsequently. Following a similar aforementioned principle, the walker executes one further step forward, reaching station III by stepping on rows C and D. At this station, the CD spectrum in principle should exhibit no spectral features in that the walker should nominally stand along the central axis of the stator, forming an achiral configuration. However, as presented by the brown curve in Fig. 2a, a slight right-handed preference is visible in the CD spectrum. This is possibly due to the assembly imperfection in the experiment as a minute deviation of the walker from the central axis of the stator can lead to immediate CD signals resulting from the high sensitivity of CD spectroscopy. Sequential addition of corresponding blocking and removal strands enables progressive walking further towards stations IV and V. The walker enters the right-handed configuration region. As shown in Fig. 2b, when the walker strides from station III to IV and subsequently to V, the CD response strengthens successively. At station V, the CD response reaches approximately −200 mdeg, exhibiting a dip-to-peak line shape, which is nearly a mirror image of the CD spectrum at station I. This importantly indicates that in the solution the walkers that were directed to walk from station I have nearly all successfully reached station V, demonstrating the high fidelity of the walking process. In short, the individual steps of the walker that are well below the optical diffraction limit can be optically discriminated in real time.

### Theoretical calculations and TEM characterizations

**Figure 3:**
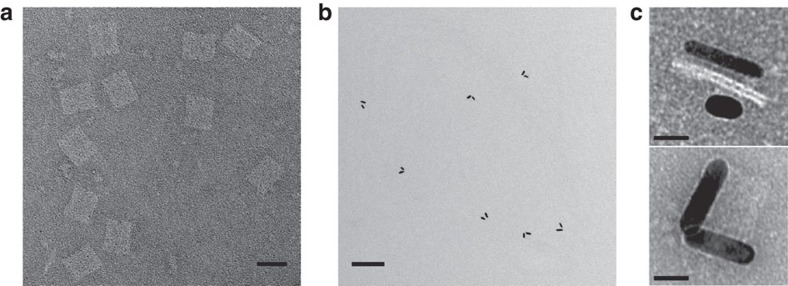
TEM images of the DNA origami templates and the plasmonic walker structures. (**a**) TEM image of the double-layer DNA origami (58 × 42 × 7 nm) after negative staining, Scale bar, 50 nm. (**b**) Exemplary TEM image of the plasmonic walker structures at station I. In the individual structures, two AuNRs (35 × 10 nm) are assembled on one origami template. The plasmonic structures display certain deformation due to the drying process on the TEM grids. Scale bar, 200 nm. (**c**) Enlarged views of the plasmonic walker structures from different perspectives. Scale bars, 20 nm.

For comparison, theoretical calculations^25^ of the CD spectra were carried out and are presented in Fig. 2c (details can be found in Supplementary Figs 2–5). In the calculations, the right-handed preference at station III was not included. Overall, the experimental spectra agree well with the theoretical results. In addition, to assess the assembled nanostructures, transmission electron microscopy (TEM) was performed. TEM images of the DNA origami templates and exemplary structures at station I are shown in Fig. 3a,b, where the rectangular origami and the formation of AuNR dimers are clearly visible. Enlarged TEM images of the AuNRs assembled on the origami from different perspectives are presented in Fig. 3c. TEM images of the structures at other stations can be found in Supplementary Fig. 6.

### *In situ* optically monitoring the walking process

**Figure 4:**
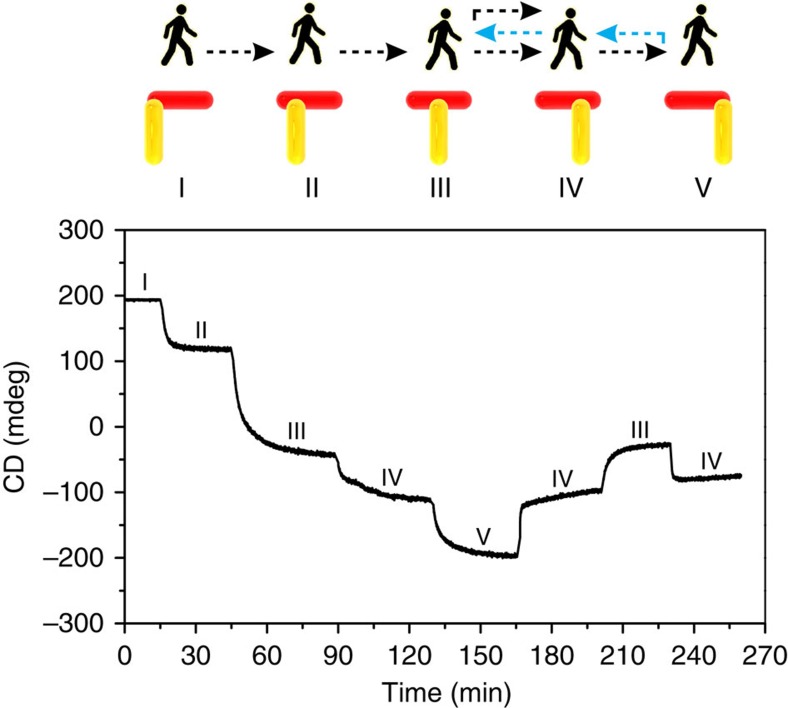
Directional and progressive walking of the plasmonic walker detected by *in situ* CD spectroscopy. The CD intensity was monitored at a fixed wavelength of 685 nm, while the walker performs stepwise walking, following a route I–II–III–IV–V–IV–III–IV.

To *in situ* monitor the dynamic walking process, CD spectra of the sample were recorded using a time-scan function of the CD spectrometer at a fixed wavelength of 685 nm. As shown in Fig. 4, the CD intensity displays a successive decrease when the walker executes discrete steps from station I to station V. In average, the transition between different steps takes ∼25 min to complete. Previous DNA walkers based on ‘burnt bridge' render impossible reverse walking along the same track^4^,^8^,^11^. To demonstrate the switchable directionality of our walker, reverse walking is carried out after the walker reaches station V. On addition of blocking strand *f* and removal strand 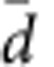, the walker changes its walking direction and executes one step back towards station VI, stepping on rows D and E. This gives rise to an instant CD intensity increase as shown in Fig. 4. When the walker executes one more step backward, the CD intensity shows a further increase to the level at station III. Subsequently, the walker makes a new turn at station III and undergoes another reverse walking towards station IV. As shown in Fig. 4, the CD intensity changes approximately back to the level at station IV. Overall, the walker has successfully carried out directed movements along the track, following a regulated route of I–II–III–IV–V–IV–III–IV.

### Stepwise walking on 3D origami

**Figure 5:**
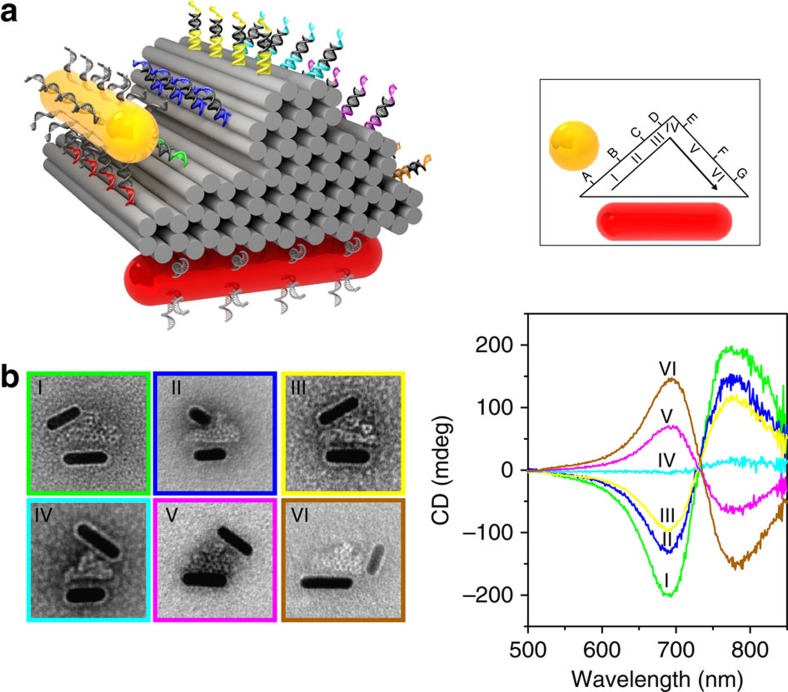
Stepwise walking on 3D DNA origami. (**a**) Schematic of the plasmonic walker on a 3D triangular prism DNA origami platform. The walking track comprises seven rows of footholds (A–G) extended from the origami surface to define six walking stations (I–VI). The walking process starts at station I, where the walker and the stator form a right-handed configuration. The distances between the neighbouring stations are slightly different owing to the irregular side surfaces of the 3D origami. The successive step sizes are ∼7, 7, 12, 12 and 11 nm. (**b**) Measured CD spectra and corresponding TEM images of the plasmonic walker structures at different stations. The frame size of each TEM image is 80 nm. The plasmonic structures display certain deformation due to the drying process on the TEM grids.

To demonstrate the capability to perform more complex behaviour, stepwise walking of the walker on a 3D origami platform is examined. Figure 5a shows the schematic of the walker system, in which triangular prism origami is utilized as the walking track. Its length is 35 nm. The three side lengths of the triangular cross-section are ∼29, 26 and 38 nm. The stator (AuNR in red) is immobilized on one side surface of the triangular prism (see Fig. 5a). Seven parallel rows of footholds are extended from the other two side surfaces to establish six walking stations I–VI. At each station, the feet of the walker (AuNR in yellow) step on two rows of the footholds. Detailed design information can be found in Supplementary Fig. 7. The stepwise walking starts from station I, where the walker and the stator form a right-handed configuration. Following the same walking strategy described in Fig. 2, the walker first carries out two discrete steps along the track, reaching stations II and III, respectively. Subsequently, the walker approaches the vertex of the triangular prism at station IV. It then makes a turn, entering the left-handed configuration region. By executing two more discrete steps, the walker eventually reaches station VI. Figure 5b shows the experimental CD spectra at different walking stations. It is evident that the individual steps along the track on the 3D origami can be correlated with distinct CD spectral changes. Representative TEM images of the AuNRs on the 3D origami at different stations are also presented in Fig. 5b. The corresponding calculated CD spectra can be found in Supplementary Fig. 8. The experimental and theoretical results show an overall good agreement.

## Discussion

The powerful combination between the precise control of nanoscale motion enabled by DNA nanotechnology and the rich spectral information offered by plasmonics^26^,^27^,^28^ suggests a new generation of artificial synthetic machines, which can *in situ* report their own structural dynamics using a noninvasive, stable and all-optical approach. This will render profound significance in dual disciplines. First, the realization of advanced walkers that can stride along multidirectional footpaths and perform different tasks on 2D or 3D prescriptive landscapes can be promptly envisioned^8^,^29^,^30^. Second, our walker concept will expand the functional scope of DNA-based devices as well as enrich the category of the state-of-the-art characterization methods for practical applications. Intriguing light–matter interaction studies, for example, distance-dependent interaction between single emitters and plasmonic nanoparticles will no longer be restricted to a static picture^31^,^32^,^33^. The plasmonic nanoparticle can be transformed to a walker by proper functionalization and prods the emitter with fully coordinated motion at the nanoscale accuracy. Finally, our walker concept also outlines an exciting prospect of generating programmable large-scale nanocircuits that incorporate biochemical, electrical and optical components for active transport and information processing.

## Methods

### Design and preparation of the DNA origami track

DNA scaffold strands (p7560) were prepared following previously described procedures. All other DNA strands were purchased from Sigma-Aldrich (high-performance liquid chromatography purification for the thiol-modified DNA and reverse-phase cartridge purification for the staple strands, capture strands, blocking strands and removal strands). Agarose for electrophoresis and SYBR Gold nucleic acid stain were purchased from Life Technologies. Uranyl formate for negative TEM staining was purchased from Polysciences, Inc.

The DNA origami track was designed using caDNAno software^34^,^35^. The two-layer track origami consists of 42 helices arranged in a ‘honeycomb' lattice. To prevent the aggregation of the DNA origami, six thymine bases were added to the respective staple strands at the edge of the origami (design and sequence details can be found in Supplementary Fig. 9 and Supplementary Table 1). The DNA origami was prepared by mixing 5 nM of the scaffold strands with 10 times of the staple strands and the respective capture strands in a buffer containing 0.5 × TE (pH=8), 12 mM MgCl_2_ and 5 mM NaCl. The mixture was then annealed as follows: 85 °C for 5 min; from 65 to 61 °C, −1 °C/5 min; from 60 to 51 °C, −1 °C/60 min; from 51 to 38 °C, −1 °C/20 min; from 37 to 26 °C, −1 °C/10 min; held at 25 °C. The DNA origami was purified by agarose gel and extracted by BioRad Freeze N Squeeze spin columns to remove the excess staple and capture strands (see Supplementary Fig. 10). The 3D origami was prepared and purified in the same way. Design and sequence details can be found in Supplementary Fig. 11 and Supplementary Table 2.

### DNA functionalization and assembly of the AuNRs

AuNRs were purchased from Nanopartz (Part# A12-10-750). Functionalization of the AuNRs with thiolated DNA (3′-SH) was carried out following a modified low pH procedure. Thiolated DNA strands were incubated with TCEP [tris(2-carboxyethyl)phosphine] for at least 1 h to reduce the disulfide bonds. The ration of DNA:TECP was 1:200. Before functionalization, the purchased AuNRs were spun down and then resuspended with nanopure H_2_O (18.2 MΩ cm) to remove excess cetyl trimethylammonium bromide. AuNRs (1 nM, 500 μl) were mixed with H_2_O (150 μl), 0.2% sodium dodecyl sulfate (SDS, 100 μl), 10 × TBE (50 μl), ∼1 M HCl (50 μl, pH=3) and 250 μM thiolated DNA strands (10 μl). NaCl (5 M, 10 μl) was added every 20 min for 9 times. Subsequently, 1 M NaOH (50 μl) was added to adjust the pH value back to ∼8. Finally, the concentration of NaCl reached 500 mM. The AuNRs functionalized with DNA were then purified to remove excess free DNA strands by centrifugation. At least five times of centrifugation steps at a rate of 8,000*g* for 30 min were carried out. Each time, the supernatant was carefully removed and then the AuNRs were resuspended in a 0.5 × TBE buffer containing 0.02% of SDS. The supernatant was then removed and the remained AuNRs were used for walker assembly on DNA origami.

The initial configuration of the walkers was left-handed. First, 10 times excess of the blocking strands c, d, e and f were added to the purified DNA origami and incubated at room temperature for 0.5 h to block the footholds C, D, E and F. The sequences of the thiolated DNA, blocking and removal strands can be found in Supplementary Table 3. Then, the purified AuNRs (both of the walkers and the stators) were added to the purified DNA origami with a ratio of five AuNRs per DNA origami structure. The mixture was incubated for over 24 h at room temperature. A second agarose gel purification step (0.5% agarose gel in a 0.5 × TBE buffer with 11 mM MgCl2) was used to purify the successfully assembled product (see Supplementary Fig. 12).

### CD characterizations

CD spectra were measured using a Jasco-815 CD Spectrometer with a Quartz SUPRASIL cuvette (path length, 10 mm). All measurements were carried out at room temperature in a buffer after agarose gel purification (0.5 × TBE buffer with 11 mM MgCl_2_, pH=8). The blocking and removal strands were prepared at a concentration of 100 μM in H_2_O for use. Generally, small amounts but high concentrations of the blocking and removal DNA strands were used for driving the walker to eliminate the dilution effect.

For individual CD spectrum measurements, the sample at the initial left-handed state was divided into five copies. All of the copies had a volume of 65 μl and respective strands were added to drive the individual systems to their designated stations. To keep the system concentration constant, equal volume of H_2_O was added, if no DNA strands were added. Four times of additions were conducted for all of the five samples. After each time of the addition, the samples were incubated at room temperature for about 1 h to ensure that they reached equilibrium (see Supplementary Table 4).

For the *in situ* CD measurements, a 400-μl solution containing ∼0.67 nM of the walkers at the initial left-handed configuration was used. The CD signal at 685 nm was monitored using the time-scan acquisition mode and a data pitch of 1 s. Respective blocking and removal strands were added to enable a programmed route. After the whole process, the total volume increased was only 12 μl (3%; see Supplementary Table 5).

### Theoretical calculations

Theoretical calculations were performed using commercial software COMSOL Multiphysics based on a finite element method. The origin of the bisignate CD in the plasmonic cross configuration is a consequence of Coulomb interaction between the dipoles of the two AuNRs. The CD signal was calculated as a difference in extinction for the left- and right-circularly polarized light. Since the plasmonic assemblies were dispersed in a solution, we carried out orientational averaging. Averaging over all possible orientations at defined light incidence is equivalent to averaging over all incident directions of light for a nanostructure with defined orientation. It has been demonstrated both analytically and numerically that averaging over six orthogonal directions of light incidence is sufficient to give accurate CD. To account for the inhomogeneous broadening arising from the polydispersity of the AuNRs, the experimental dielectric function of Au was modified by including an additional term:


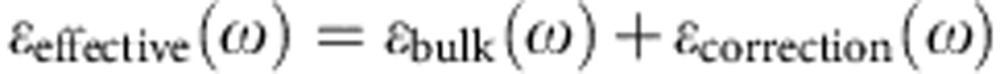


where the dielectric function of bulk Au, *ɛ*_bulk_ is from Johnson and Christy values^36^, and the correction term is introduced following a standard approach:


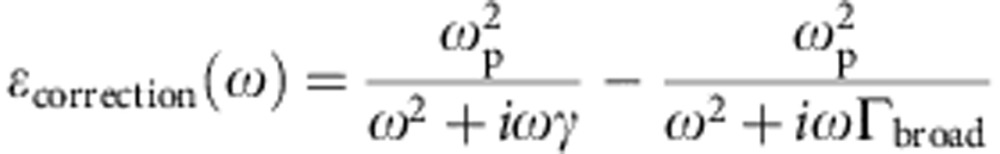


where *ω*_p_=8.754 eV and *γ*=0.0724, eV are the Drude parameters, respectively. Γ_broad_ is 0.362 eV.

## Additional information

**How to cite this article:** Zhou, C. *et al.* A plasmonic nanorod that walks on DNA origami. *Nat. Commun.* 6:8102 doi: 10.1038/ncomms9102 (2015).

## Supplementary Material

Supplementary Information Supplementary Figures 1-12, Supplementary Tables 1-5 and Supplementary Note 1
