# Supplementary material for: A plasmonic nanorod that walks on DNA origami
Source: Nat Commun. 2015 Aug 25;6:8102. doi: 10.1038/ncomms9102 (PMC4560816; doi:10.1038/ncomms9102)
Supplement: Supplementary Information — Supplementary Figures 1-12, Supplementary Tables 1-5 and Supplementary Note 1 [file ncomms9102-s1.pdf]

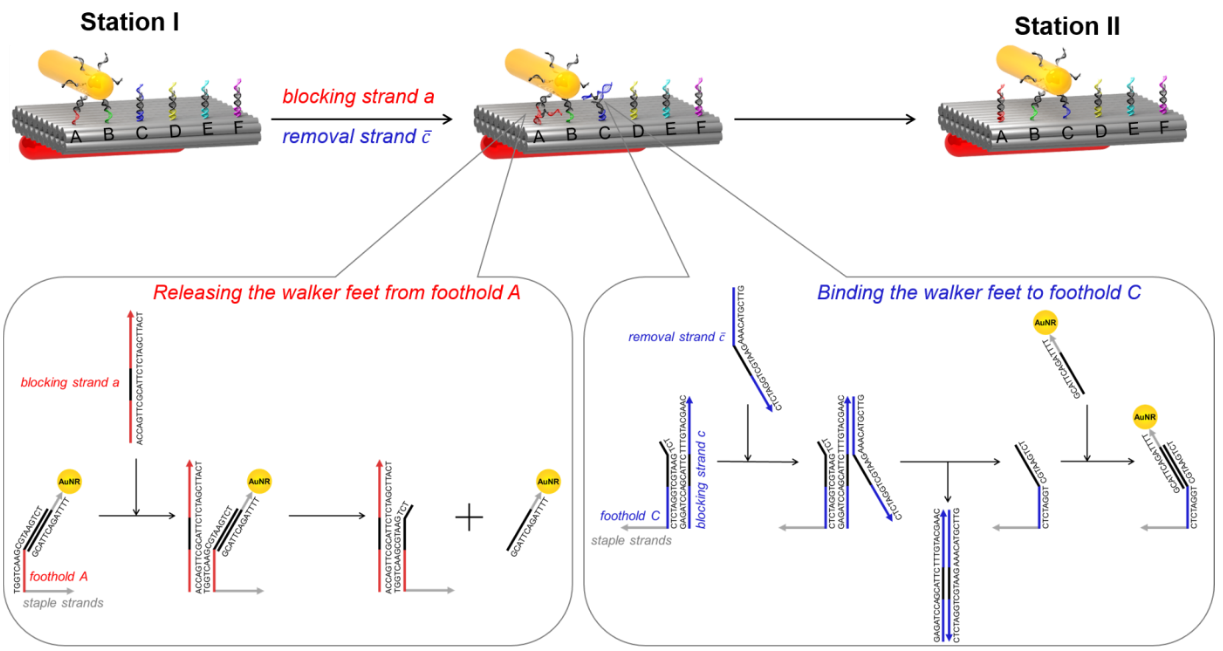

Supplementary Figure 1 | Schematic illustration of the walking process from station I to station II. Each colorful foothold strand extended from the DNA origami track represents one row (totally 5 strands) of identical footholds. At each station, the walker AuNR binds to two parallel rows of the footholds during each walking step. There is always one row of the footholds which binds to the walker to ensure progressive walking. For example, at the initial state (station I), the walker binds to rows A and B. During the walking process, the walker always binds to row B. The addition of blocking strands *a* enables the displacement of the foot strands from row A. Meanwhile, blocking strands *c* were released by their removal strands to activate the foothold C. These two processes make footholds C become accessible for the walker feet and the walker then binds to footholds C, finishing the first step. As every foothold can be reversibly blocked and activated, the track is reusable. This implies that the walker can do reversibly directional walking.

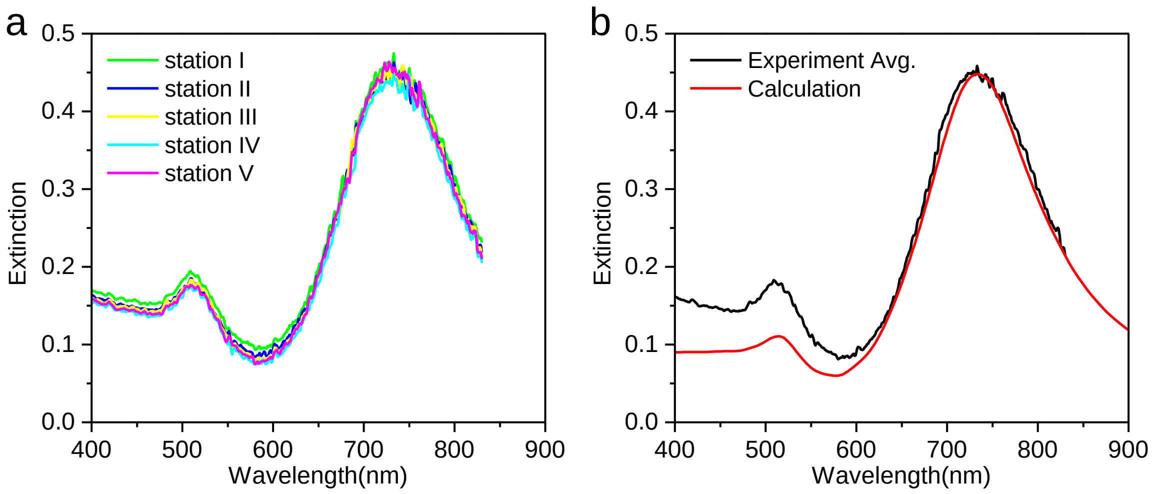

Supplementary Figure 2 | UV-VIS spectra of the walker. a. UV-VIS spectra of the sample at different stations. The transverse and longitudinal modes are at 510 nm and 730 nm, respectively. **b.** Experimental and calculated extinction spectra. Apparently, the extinction spectra cannot provide useful information on the conformational changes of the plasmonic assemblies in that the spectra display nearly the same profile at different stations. The concentration of the AuNRs in the sample can be estimated to be 0.67 nM based on the absorption. It is worth mentioning that in the CD calculation, we assumed that all AuNRs were assembled on origami, that is, an assembly yield of 100%. However, in the experiment the assembly yield cannot be ideal. This explains the intensity difference between the experimental and calculated CD. The dimension of the AuNRs is estimated to be 35 nm×10nm. The vertical distance between the two AuNRs is estimated to be 18 nm according to the DNA structure.

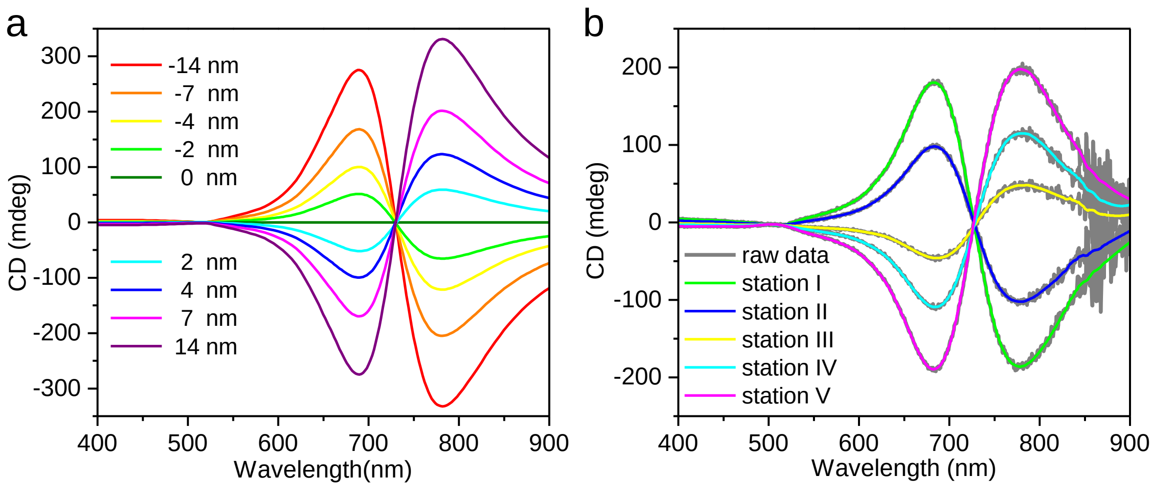

Supplementary Figure 3 | Calculated and experimental CD spectra as a function of the waking distance. To achieve insight into the walking process, CD spectra as a function of walking distance were calculated (a). The experimental CD spectra (b) are shown for comparison. Stations I, II, III, IV, and V correspond to the displacements of -14 nm, -7 nm, 0 nm, 7 nm, and 14 nm. Each step size of the walker is 7 nm. The agreement between the experimental and calculated CD spectra is very good.

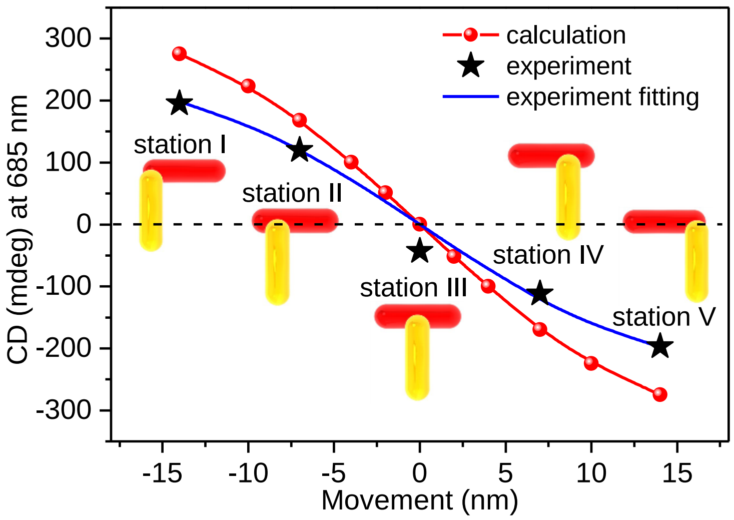

Supplementary Figure 4 | Experimental and calculated CD-Ms. In order to quantitatively describe the sensitivity of the plasmonic walker, a figure of merit of a chiral plasmonic walker is introduced as "CD (mdeg)/walking distance (nm)" (CD-M), that is, the ratio between the CD intensity at a signature wavelength and the walking distance. The experimental and calculated CD-Ms are 13.6 mdeg/nm and 19.5 mdeg/nm, respectively. This implies that the CD intensity can change as large as 13.6 mdeg per nm, ensuring optical probing of the dynamic process with high sensitivity.

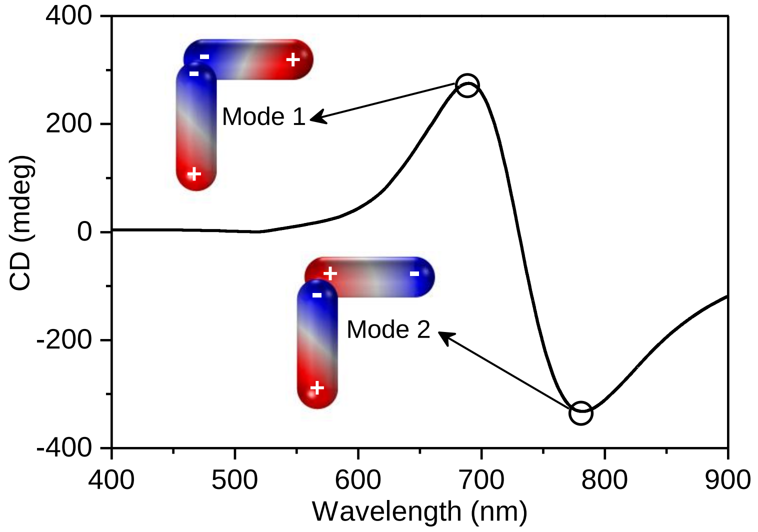

Supplementary Figure 5 | Charge distributions at the two different modes. To understand the underlying physics, the charge distributions at different resonances were calculated. The CD spectrum as station I is used exemplarily. It shows the characteristic peak-to-dip line shape. Mode 1 at 685 nm corresponds to the symmetric mode excitation, in which the charges oscillate in phase in the two AuNRs. Mode 2 at 780 nm correspond to the anti-symmetric mode excitation, in which the charges oscillate anti-phase in the two AuNRs.

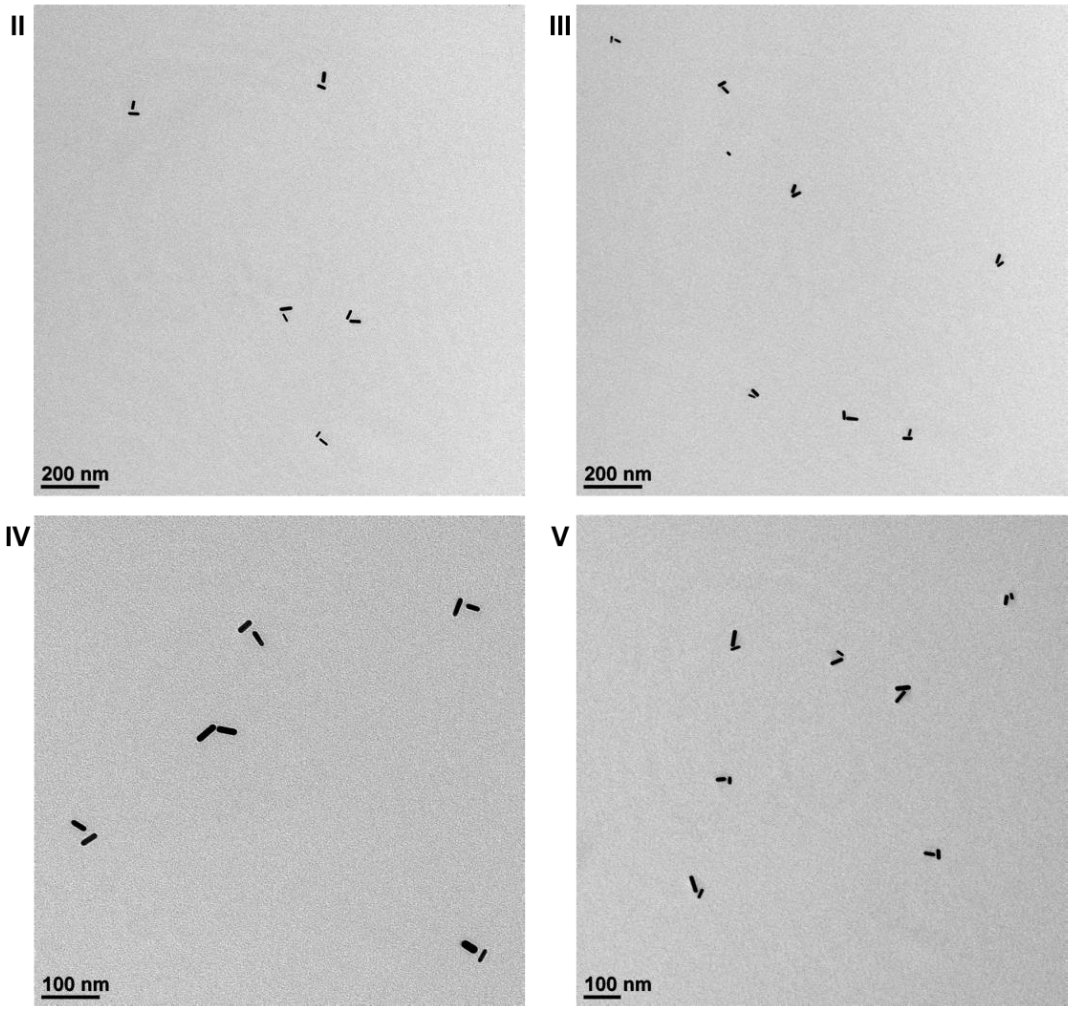

Supplementary Figure 6 | TEM images of the plasmonic walker structures at stations II, III, IV, and V. Due to structural deformation on the TEM grids resulting from the drying process, the 3D conformation changes of the structures at different stations cannot be clearly distinguished using standard TEM spectroscopy. However, the 3D conformation changes of the structures at different stations can be clearly discriminated using CD spectroscopy optically.

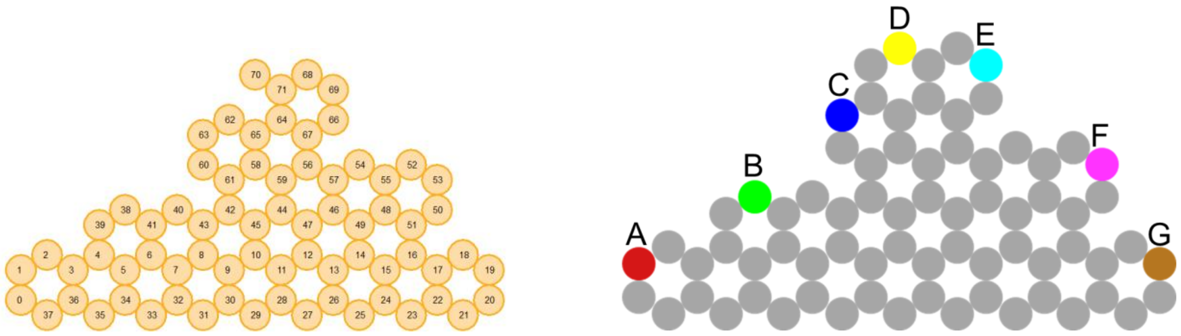

Supplementary Figure 7 | Helix numbers of the 3D DNA origami and the arrangement of the footholds on the origami.

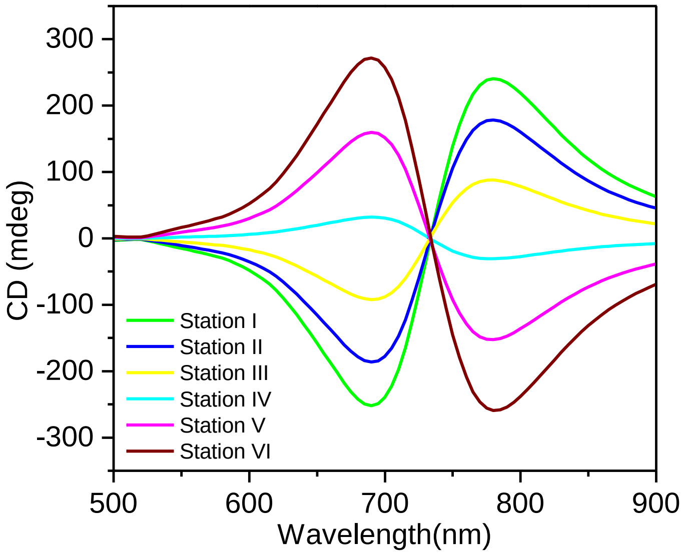

Supplementary Figure 8 | Theoretical calculations of the walker on the 3D DNA origami.

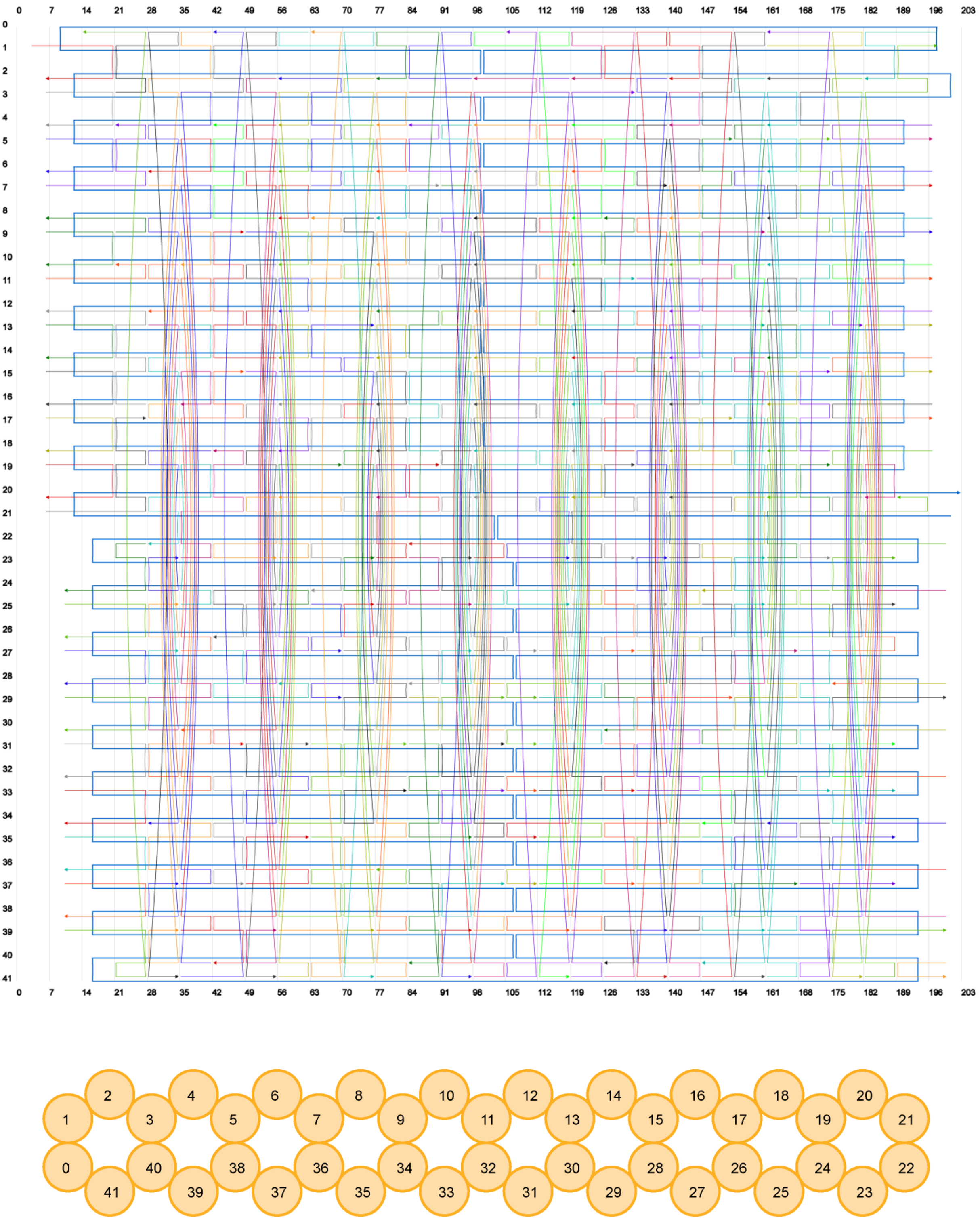

Supplementary Figure 9 | Strand routing diagram of the two-layer plate DNA origami track.

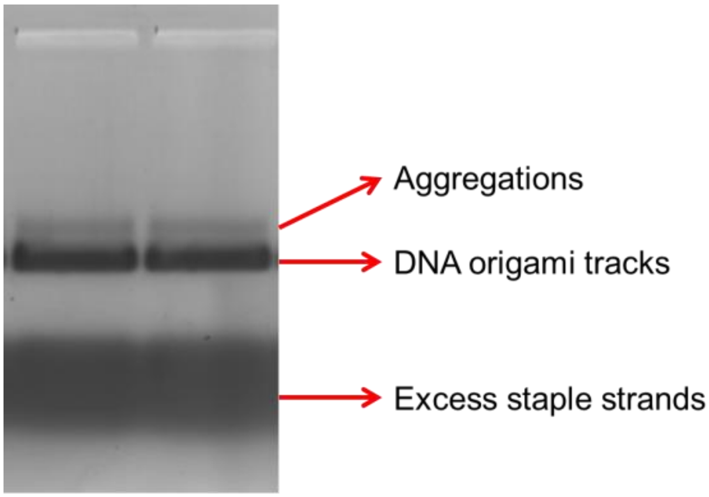

Supplementary Figure 10 | Inverted photograph of a SYBRGold stained 0.7% agarose gel under blue light (460 nm) illumination. The DNA origami template structures were purified to remove excess staple strands.

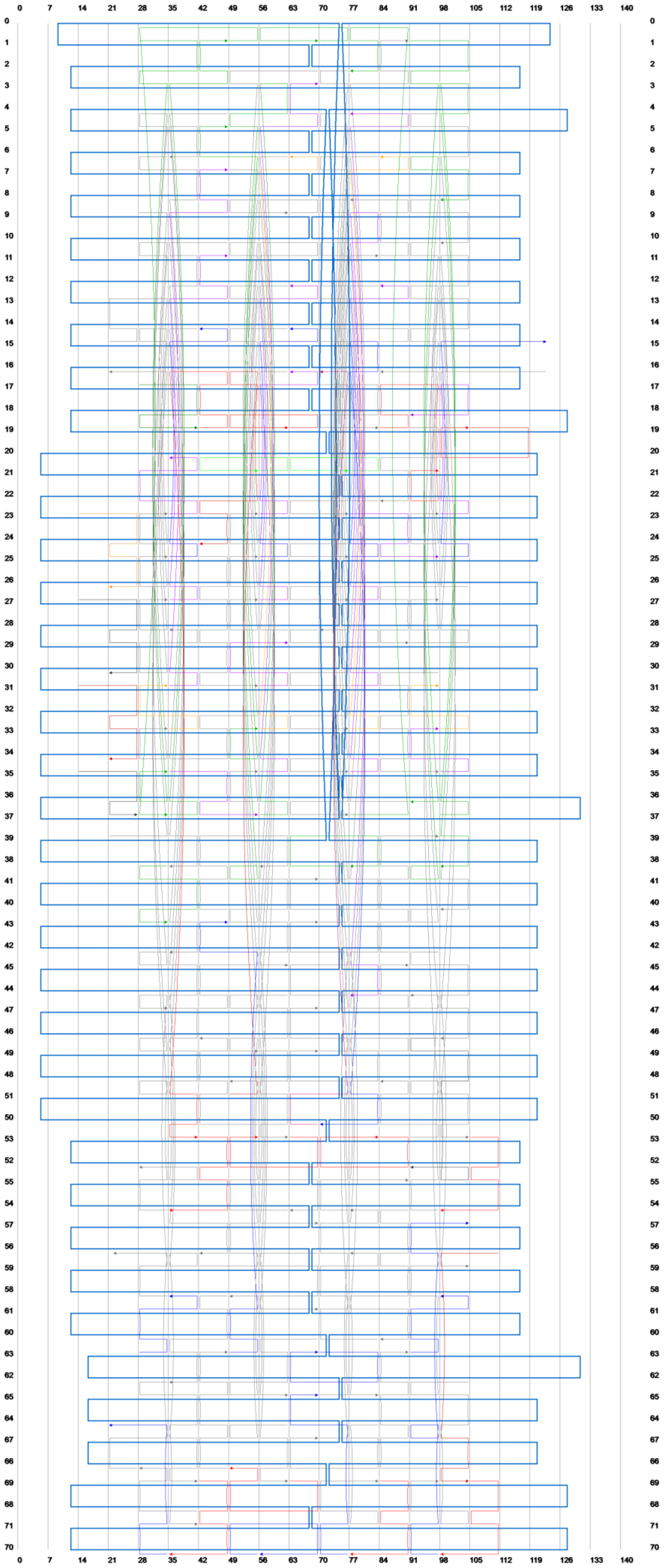

Supplementary Figure 11 | Design of the 3D DNA origami track.

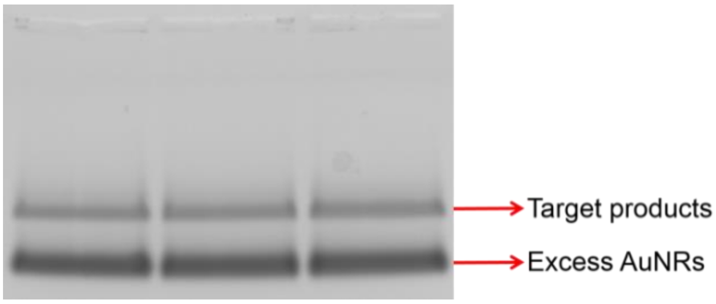

Supplementary Figure 12 | White light image of 0.5% agarose gel for separating the target plasmonic walker products from the excess AuNRs.

Supplementary Table 1. Sequences of the DNA origami track

Start	End	Sequence	Length
Core staple strands			
25[119]	20[119]	CAACATACGGGGAGCGAACGTACCCTAA	28
33[112]	12[119]	GCGCATCGTAACCGAAGGGCGCTGAGAGAGAGGTG	35
23[84]	25[97]	AATTTTCTTTCAGCAAACAGCTTGATAC	28
23[77]	19[90]	GTAAATGCCAATAGCCGCCACCCTCAGAGAAGGATATTAAGA	42
7[6]	4[21]	TTTTTTTAACGTCAAAAATGAAGAAACGAAGGTTTTTTTAGCG	43
41[98]	2[98]	CAATTCTGTTTTAAGATGTGAGTGAATAGCTGATGCAAATTG	42
2[55]	39[55]	CCATCCTAGTACCGAACAGTTAGGTCAT	28
12[139]	29[153]	GCAGAAGAAGCGTACTTCTAAACCTTATGACAATGTCCCGCC	42
35[63]	6[56]	AAAACCACAATACTCCTGACTTAATTCGAGCTTCATTTGCACATAAACA	49
37[35]	34[28]	GACCATAATTGAATGCCTTTAGAGTTAAGCCCAATCTATCTTCCAAAAG	49
29[10]	30[10]	TTTTTTCCCCCAGCGATTCGGTGTACAGACTTTTTT	36
27[112]	18[119]	TCACATAAATCATTTGTGAAAGGAGCTATTTGACG	35
14[196]	28[175]	TTTTTTAAATACCTACATTTTGGGAAAAATACCGAC	36
27[10]	28[10]	TTTTTTGTCACCCTCAGCACTCATCTTTGATTTTTT	36
25[77]	20[77]	ATTTCTTGGAGTGATGAAAGTTAGGATT	28
23[119]	19[132]	GGGTGGTAAATCCCTGAGTGTTGTTCCAAATCGGAGGCGAGA	42
16[160]	25[160]	CTGTCCAACAGGAACTCGAATGGGGTGC	28
0[62]	41[76]	GCCATATGACGACGACAATAAACAACATCTTAATTTTTGGGG	42
20[181]	23[174]	ACCATCAGTCAAAGCGCTGGTTTGCCCCCACCGCC	35
36[104]	6[98]	GGGGTAACCTGATGGCAATTCCGGGAGAAACAACG	35
37[168]	0[161]	GTCAATCCGTTCTAAGAAAGGAACATTAAAGCAATAATAAACACCGGAA	49
41[140]	4[140]	ACAGGCAGGAGAAGATTAATTTCATTTG	28
20[97]	20[98]	CTCAAGAGCCACCACCCTCTAGCCCGAGCCCCGATTTAGATC	42
8[118]	35[111]	CATATTCAATACATCACGTTGGTGTAATAGTTTGT	35
21[6]	17[27]	TTTTTTGAATAGGTGTATCACCATATAAGGCCCGTACGGGGTCTGAGGCA	50
30[181]	29[199]	GGCCAGTTATTTACGCTCGCCCTGGATTTTTT	32
18[132]	14[119]	TGGTTGCAACAGGAGCAATACGGCCTTGACCAGTCACACGAC	42
23[56]	20[56]	TTGTCGTTAACACTTCAGAACCGCCACCTTTTGCTCAGTACC	42
20[55]	23[55]	AGGCGGAGCCACCCGAGTTTCGTCACCAAACGATCTAAAGTT	42
1[161]	41[181]	TCTGACCTAAATTTAATGGTTAAATAAGAAAGCCT	35
18[196]	23[188]	TTTTTTCGCTGCGCGTAACCACGCAAGTGCGCTTTCTGGCCCTGAGAGAG	50
6[76]	8[77]	TTGCCAGCGCATTAGACGGGAGGTAATT	28
6[55]	35[62]	GCCATATAATAACACGTCATAACCAGACGACGATA	35
27[98]	16[98]	AGGAAGTCCGACAATCCCTCGTTAGAATATAACATCACTTAG	42
12[76]	8[63]	CATTCAACCGATTGATTTTGTCTGGCATAGATAGCAATATCA	42
35[10]	36[10]	TTTTTTATAGTAAGAGCAAAATGCTTTAAATTTTTT	36
16[27]	12[6]	GCCGCCGTAGCCCCGCGTTTTGACTTGATTAAAGGTGAATTATCTTTTTT	50
2[97]	41[97]	TTTATCAACAATAGTAATGCAGAACGCGAGCCAACGTGGCAT	42
25[63]	27[69]	TGCTTTCGATATATCTACAGA	21
39[10]	0[14]	TTTTTTTAGAGAGTACCTGTAGATTATTAGATGAGGCATTTTCGAG	46
30[76]	16[77]	TCAATCAGCCTGATAAATTGTATGCCACCCACCACGAACCGC	42
13[6]	14[6]	TTTTTTACCGTCACCCATCGGCATTTTTTT	30
41[126]	2[119]	ATCCAATTATCATATGCGTTACGCGAGAAACTATA	35
18[41]	23[34]	GTAATAAGTTTTAATAAACAGTTCACGTCTCATAG	35

25[161]	20[161]	CTAATGAGAAACCTGCGCTAGCAAGTTT	28
1[3]	2[6]	TTTTTTAATAAGAGAATATAATCGGCTTTTTTT	33
22[199]	20[189]	TTTTTTGCAAGCGGTCCAGGCGAAAAACCGTCGGCCCAC	39
34[199]	9[196]	TTTTTTATTAAATGTGAGGAACGTTATTTTTTT	33
9[21]	8[6]	GCAATAGAATAAGAGCAAGAAACTTTTTT	29
2[76]	39[76]	CTGAACAAAGCAAGTCTGGAATAGAGCT	28
13[35]	27[34]	GGGAATTCGTCAGACTGTAGCCTTATTACTAAAACAGCGAAA	42
3[84]	39[97]	TATTTTCATCGTAGATATGCAAATGCTG	28
15[6]	16[6]	TTTTTTTTTCGGTCACCAGCATTGTTTTTT	30
41[147]	2[140]	AGGCAAATACTAGAAAAAGCCATATTTTCCGGCTT	35
37[112]	8[119]	CCTGAGAGTCTGGATTAAAATTAATCCTTTATCAT	35
2[160]	40[147]	TACCTTTAGAATCCGTAATACTTTTGCG	28
29[112]	16[119]	GTCCTTAGTGCTGAGGGCCTTAACTATCTTCTTTG	35
35[147]	39[160]	TCAAAAATATTTAACGGTAATGCCGGAGAGGTAAAGATTCAA	42
25[98]	18[98]	CGATAGTACAACTTACGCTTGACGGGGAATAACGTGCTTTTG	42
34[160]	12[161]	CTCCGTGCAGTATCGGCCTCAAGGCAAACTGAACCGCCATTA	42
4[174]	2[161]	AAAACAACATAGCGTAGGTCTGAGAGAC	28
17[6]	18[6]	TTTTTTACAGGAGGTAGTGCCTTGTTTTTT	30
23[161]	19[174]	TGCCCTTAGCAGGCACGTGGACTCCAACCCCAAATGGCGCTG	42
6[160]	34[161]	TTTTCAGGGAAGGGAAGCAAATAATTCGCGTCTGGTCGGATT	42
31[112]	11[132]	CGCTATTACGCCAGTGAATTCCCCTTCTGACCTGAATAAAACCCAGCAG	49
7[126]	4[119]	GATTGTTCAGTAACTACCAAGTTTTTTAATGGAAA	35
25[10]	26[10]	TTTTTTAAAAAAAGGCTCTTTTGCGGGATCTTTTTT	36
31[126]	33[132]	CTGGCGATGTTGGGTGCATCT	21
30[69]	14[56]	TAAGGGAACCGAACCACCAGTCAGAATC	28
4[153]	41[160]	AACAATTTTCCCTTTTAACCTAGTTAATTTCATCTTCATAATGAATTAG	49
31[10]	32[10]	TTTTTTCAGGCGCATAGGATCATTGTGAATTTTTTT	36
20[118]	23[118]	AGGGAGCATAGGGTTTATAAATCAAAAGCTTATTGGGCGCCA	42
5[6]	6[6]	TTTTTTACTTGCGGGTTTTTTGTTTTTTTT	30
28[104]	14[98]	ATTAAACTCGCCTGAGTAGAAAAAAGGGACATTAT	35
3[6]	4[6]	TTTTTTGTCTTTCCTAACCTCCCGTTTTTT	30
24[132]	27[132]	CAACGCGCGAGCCGTTTCCTGTCTCCGA	28
41[84]	2[77]	TGAAAAGGCTCAACAGTAGGGGTTCAGCATAAGTC	35
30[153]	14[161]	CCAGGGTGCAAGGCGATTAAGTTGGGTATTTCTCAATTTTTGATCGTCT	49
8[139]	37[132]	CAGAAGGGTATTAGATTGACCGTAATGGTTTTAACTATTTTGGCAAACA	49
30[160]	16[161]	GGAGAAGAAAATAACCCCGCTCCCTGCCCAGCCATAAAGAGT	42
4[196]	37[188]	TTTTTTTGAAACAAACATCAAGGAGCAAAATTCAACATATGTACCCCGGT	50
27[70]	18[77]	GGCTTTGAGGACTAGCCCACGGCCAGAAGTAAGCG	35
33[10]	34[10]	TTTTTTTACCTTATGCGAGAATTACGAGGCTTTTTT	36
16[118]	27[111]	ATTAGTACAGAGCGTTGTTATCCGCTCGTTGCATT	35
16[153]	13[160]	TCACGCAAACAATAATTATTTGTGGCACAGACAAT	35
7[28]	10[35]	AATAGCACCCCCTCACACTATCATAACCCATAACGACCGAAGAGTATGT	49
11[133]	14[140]	CAAATGAGCGCAACAAGGGGGATGTGCTGGATGTTAGAATACACATTGG	49
41[35]	0[42]	GCAAATGGTCAATACGCCAAC	21
16[196]	25[188]	TTTTTTATAATCAGTGAGGCCACCTGAGAGGAGGATAACTCACATTAATT	50
12[196]	13[181]	TTTTTTCGAACTGATAGCCCTAATTAGTC	29
27[42]	28[56]	TCGGAACGGCAAAAACAAAGTACAACGGAGATTTGCCTAAAA	42

15[49]	18[56]	TTCATAACGAAAGAGAGGGTAGCAACGGTCGGTCGAACAAATTGATGAT	49
23[98]	22[84]	GATTTTGAAATTTTCAGGGATAGCAAGC	28
37[49]	8[56]	AATCAGGTCTTTACGCGGAATTAAAAACGAGAGAT	35
10[160]	34[147]	AAGGAATCACCTTGGCGCCATTCGCCATACGACGAGGAACAA	42
16[34]	25[34]	CACCAGAGGTCAGAAGGCCGCCAAAAGG	28
23[35]	18[42]	TTAGCGTGTACAAAGGAGGTTTAGTACCTAAGTGCCCCCTGCTGTACTG	49
35[98]	8[98]	TTTTGCCTATTACAGACCGTCAATAGATCTGATTATCAGACT	42
9[49]	12[56]	TAAGAAAAACTAATACCAGTCAGGACGTTAAATTGTAAGTTTAGGGAGG	49
40[41]	37[34]	AATTCTGCGGGTATAGAACGCGAGGCGTGAAGCCTAACTCCACGAGAAT	49
28[199]	15[196]	TTTTTTGTGACTCTATGACGCTCATGGTTTTTT	33
11[6]	10[21]	TTTTTTATAAAGGTGGCAACATCGTAGAA	29
38[199]	5[196]	TTTTTTCATCAATATGATAGAAGATGATTTTTT	33
0[197]	2[182]	TGATAAATAAGGCGTTTGAAATAATTTATC	30
25[56]	19[69]	ATCAGCTAACTAAAGGAACCTATTATTC	28
27[168]	33[174]	GGCTTAAAGTGCGGTCTAATCGCCAAGCACGCCAGGGAAACCGGAAGAT	49
27[35]	15[48]	GACAGCAGAGTTAACGATTGGCCTTGATGCCACCACCATCTT	42
10[196]	31[188]	TTTTTTTATCTGGTCAGTTGGCTCAAACCTGGTGCCGGTTTTCCCAGTCA	50
23[140]	20[140]	GTGAGACATGGTGGACAAGAGTCCACTATCGAGGTGCCGTAA	42
31[84]	35[97]	TCAACGTCCCTGACATAAAACAGATTCATTTTGCAAAAGAAG	42
25[147]	16[140]	AAAGCCTTCGTAATGTTGGTGAGCAACTCGTCGGTTATCCAGAATTAAC	49
39[119]	6[119]	TTTTAAATCTACAACTTTGAAAGTACCT	28
18[69]	16[56]	TGGCTTTAAATCCTAGAGCCACCACCCT	28
16[97]	28[84]	AGCCGCCCGCAGTCTGACAACAACCATCAAGACTTATACGTA	42
10[97]	31[104]	CGCAATAATGGTTTAGGCTTGAACAAAGCTGCTCA	35
30[104]	12[98]	TTACTTACACTGGCCAACAGACAGTATTAACACCA	35
8[196]	33[188]	TTTTTTTAATTTTAAAAGTTTGTTTGCCCCGAGTAACGCACTCCAGCCAG	50
33[133]	12[140]	GCCAGTTTGAGGGGTCAGGCTAAAATCTACCACCA	35
34[174]	8[161]	CAACCCGCCTTCCTCAGGAAGATTGTATTTAGAACTTATCAT	42
7[63]	2[56]	AGGGAAGTTACAAACCAGCTAAGATATACGAGAACAGAAAAATAATATC	49
16[76]	25[76]	CACCCTCCATTAAACATAACCGAGGTGA	28
3[42]	41[55]	TAAACCAAATTTACAGTAATTCTGTCCATTAACAAACCTGTT	42
19[6]	20[6]	TTTTTTAGTAACAGTTATAGCCCGTTTTTT	30
32[104]	10[98]	GTGAATAACCGCCTGCAACAGAATAGATTAGAGAA	35
20[139]	23[132]	AGCACTAGTTTGGATTCCGAAATCGGCATTTTCTT	35
14[55]	9[48]	AAGTTTGCCTTTAGAGAGCCAAATATTGAAGACACATTACGCCCCTTTT	49
25[35]	22[28]	AGCCTTTTAATTTTTTAATGCCGTCGAGAGGGTTGGTACTCACTACAAC	49
7[91]	40[84]	CTGAACATAGTAAAATGTTTACAAAGCGAAGACTTTGAATATACTAAAG	49
18[174]	15[174]	CACACCCCCAGAATCCGAGTATGCAACA	28
2[118]	0[105]	TGTAAATACCTTGCAAAAATTTTTAGATACTAATATCTTACC	42
30[199]	13[196]	TTTTTTTTGTAAAACGACTTTAATGCGTTTTTT	33
3[21]	41[34]	TATCATTCAATCAATAAAGTACCGACAATTAGGCAACATTTC	42
20[160]	24[147]	TTTGGGGTTAAAGAGAAAATCCTGTTTGGGGCAACCAGCTGC	42
18[90]	27[97]	CGTTCCATGGAAAGACCCTCACGGAACCGCCTCCCGGGTAAATTTCATG	49
2[132]	41[139]	GTTATATAAACTTTTTCAAATTGTTTAGAAATCAT	35
22[27]	24[10]	GCCTGTAGACAGCCTGAAAATCTCCATTTTTT	32
20[76]	24[63]	AGCGGGGCTCAGAAGAACCCATGTACCGCTTTCCAAAGGAAC	42
2[139]	39[139]	AGGTTGGGCTATTACCTTTATCCTGAGT	28

0[41]	39[34]	ATGTAATAAGGTAAGAGCATGTAGAAACCCAAGAACGAACGATTAATTG	49
24[199]	20[182]	TTTTTTGCGCTCACTGCCTAGCGGTTACGTGA	32
37[133]	8[140]	AGAGAATCGATGAAATTGTAATACTTCTAAACCAC	35
12[118]	31[111]	AGGCGGTGATAGAAATGCGCACGACTTGTTCTCTT	35
25[140]	17[153]	AAAGTGTATTAATGGGAAGAAAGCGAAAGCCGCTAATTTTAG	42
10[55]	31[62]	ACTCCTTCACGGAAGGCTTGATAATCTTGACAAGA	35
29[84]	14[77]	GTCGAAACGAGGCGCAGACGGTAGCAAGCAGCACC	35
36[199]	7[196]	TTTTTTATCAGAAAAGCCTTATTTGCATTTTTT	33
40[199]	41[199]	TTTTTTCTAAATAAAGTTTTTT	22
39[35]	6[28]	CTCCTTTGGAAGCATAAATCACCCAATCCAAATAA	35
2[181]	39[199]	AAAATCAATAGCTTTACCAAACCGGAGACAGTCAAATCACTTTTTT	46
36[139]	10[140]	ACGTTAACAATAGGAACGCCAACGGCGGACTTTACGGTTATC	42
15[56]	13[76]	TCAAAATCACCGGAGTAGCGAAGCACCATTACCAT	35
13[28]	16[35]	GCCATTTAGATGAAATACCAAGCGCGAAGAATACAGCGTTTGGAACCAC	49
24[174]	18[161]	CAGTCGGGTGAGCTCCCCGGGTACCGAGCGGTACGGCCGCGC	42
32[48]	37[48]	GATGGTTTCATTATGCAGATACTCGTTTAATATTCAATCAAA	42
41[182]	1[197]	CAGAGCATCGGTTGAGATTAAGACGCTGATAGTGACCGACCGTG	44
10[34]	30[35]	TAGCAAAATAAAAGAACTTTACTGGCTGACCTTCAAGAGGAC	42
31[63]	10[56]	ACCGGATCGAGTAGTGGGAAGGGAATACCACATTCAGTAAGCGATTAAG	49
32[83]	7[90]	GAGAAACAATAGAAAATTCATATAACGGAGAAGGAGTCAGAGGAATTAA	49
14[174]	10[161]	ACGCTCAAATGGCTAAACATCTCAAATAAAATCAACAGTTGA	42
35[112]	10[119]	TAAATCAGCTCATTGATAGGTTTGAGGAGAGCACT	35
0[104]	41[118]	AGTATAACCCCAATCGCAAGACAAAGAATACAAATGTAGTAG	42
27[133]	18[140]	ACTCTGACCTCCTGCATGGTCTAAAGGGCAGGGCG	35
32[199]	11[196]	TTTTTTCGGCACCGCTTCCTCAATCAATTTTTT	33
9[6]	10[6]	TTTTTTAATGAAATAAATACATACTTTTTT	30
6[97]	4[84]	AGCGTCTTTCCAGAATCTTACGCGCCCA	28
9[161]	5[174]	CTCGTATTAAATCCAGTAACACTACCATAGAAATTATTACCT	42
37[10]	38[10]	TTTTTTCAGTTCAGAAAAACAGGTCAGGATTTTTTT	36
27[175]	17[196]	GCTACGTGGTGCTTTAAAGACAGTGTTTTTTTTTTT	36
33[28]	12[28]	TTTTAAGAACTGGCTAATTTCAAACGCAACGGAAATTATTCA	42
36[76]	10[77]	TAGCGTCAAATAGCGAGAGGCTCAGTTGAGTTACCAATACCC	42
39[77]	6[77]	TAATTGCCAAATATATCCTGAGCCTAAT	28
6[196]	35[188]	TTTTTTCGTAAAACAGAAATAAATCAAAACCAAAAAGTAGCCAGCTTTCA	50
Capture strands for the stator AuNR			
39[56]	0[63]	ACAACAACATTTTGCGGATGGCTGTTTCATATTTTCAGAGAATC	44
41[56]	4[56]	ACAACAACATAGCTATTCCATATCACTCATGAAGGCT	37
41[77]	4[77]	ACAACAACACGCGAGCTACGGTGCCGTTTTATAGCAA	37
39[98]	4[98]	ACAACAACATAGCTCAAAGAGGAAATAACGGATTCGCTAAATCAATATAAT	51
41[119]	3[132]	ACAACAACACATTAACCAAGGATTTCTGTAAATCGTC	37
39[140]	7[139]	ACAACAACAAATGTGTAGGGTAGATCGCGCGTCAGATGAATATATGGATTA	51
39[161]	6[161]	ACAACAACAAAGGGTGGCTGATACATTTCAGCGTAGA	37
41[161]	4[161]	ACAACAACACAAAATTTGACCCTTTGAAAAAATTAAT	37
Staple strands in foothold row A			
4[55]	40[42]	TCTGAATGCGAACTGGTTATCCGGTTGCTATAAGCGAACCAGACCTGATAAG GATTCCC	59
8[55]	4[42]	TCTGAATGCGAACTGGTAACCCACAAGAATTCAGAGAGTATTTATAGATTA GTATTCTA	59

12[55]	31[48]	TCTGAATGC GAACTGGTGAAGGTAGCAAAATTGACCAACTTTGAATCAAGAG	52
16[55]	25[55]	TCTGAATGCGAACTGGTCAGAGCCATTCACACTGAGGCTCGGTTT	45
18[55]	26[42]	TCTGAATGCGAACTGGTACAGGAGCTATTTCGGAATTGCGAATAAAATTGTATTGCAGG	59
Staple strands in foothold row B			
4[76]	36[77]	TCTGAATGCATGCAAGGGCAAATCCAATTTTCGCGTTTATTATAGTCAGAAGGACTGGA	59
8[76]	32[77]	TCTGAATGCATGCAAGGGAGCGCTCGAACAAAGATTTAAAAAATCTACGTTA	52
10[76]	31[83]	TCTGAATGCATGCAAGGAAAAGAACACAATCACCAGAAATTCATTACCCAAA	52
14[76]	29[69]	TCTGAATGCATGCAAGGGTAATCAACCAGAGTACGAAGGCACCAATATCATC	52
18[76]	23[76]	TCTGAATGCATGCAAGGTCATACATGAAACAGAATAGAGACGTTA	45
Staple strands in foothold row C			
4[97]	37[104]	TCTGAATGCTGGATCTCCATTACCCAACGCTAGCCCGAGATTGCATCAAAAA	52
8[97]	33[104]	TCTGAATGCTGGATCTCGAACAAAAACCGAGGGTAGAAGAACTAACGGAACA	52
12[97]	29[104]	TCTGAATGCTGGATCTCGCGCCAAACGTCACGCCGGAATCCGCGACCTGCTC	52
14[97]	12[77]	TCTGAATGCTGGATCTCGAAACCATCGATAGGCCGGAAAGACAAAAGGGCGA	52
18[97]	23[97]	TCTGAATGCTGGATCTCAATTTACGGCTGAGTCAACAGTGTATGG	45
Staple strands in foothold row D			
4[118]	39[118]	TCTGAATGCGACTCTAACAGTACACTGATTGAGGCTATCAGGTTTACAACCCTCATATA	59
6[118]	37[111]	TCTGAATGCGACTCTAATTTACATATCAATATCGCATTAAATTAGGACATTG	52
10[118]	33[111]	TCTGAATGCGACTCTAAAACAACTTGCCACGATCGGTGCGGGCCAACGATGG	52
14[118]	29[111]	TCTGAATGCGACTCTAACAGTAATGAACTCAGAATCGGCTGACCCCATAAGT	52
18[118]	25[118]	TCTGAATGCGACTCTAAAGCACGTAAGCCGGAGGCGGTTTGCGAATGCACAATTCCACA	59
Staple strands in foothold row E			
4[139]	40[126]	TCTGAATGCGGAGAGTTAATTACCTTACAAACTATTTTTGAGAGATGCAATGTTCAACG	59
10[139]	8[126]	TCTGAATGCGGAGAGTTTAAAATATCTTTAGTTTAGAAAGCGGAA	45
14[139]	30[126]	TCTGAATGCGGAGAGTTCAGATTCCTGGTAAGGGCACGAATATAGATTGTCAGTGGTTG	59
16[139]	25[139]	TCTGAATGCGGAGAGTTCGTTGTAGGCCGATATAGCTGGAAGCAT	45
18[139]	23[139]	TCTGAATGCGGAGAGTTCGTACTAAAGGAAGAATCGGCTTCACCA	45
Staple strands in foothold row F			
4[160]	37[167]	TCTGAATGCGGTCTAATTACATTTAATTATTAATTAATCGTAAAACTAGCAT	52
8[160]	5[153]	TCTGAATGCGGTCTAATTTTGCGGAACAAAGGAATAATGTTTAACAGAGGCG	52
12[160]	9[160]	TCTGAATGCGGTCTAATAAAATACCGAACGAAAAGCATTGAGGAAAAACAATTCGACAA	59
14[160]	27[167]	TCTGAATGCGGTCTAATGAAATGGTTACCGCATCTGTATAATGAGTAAACAG	52
18[160]	23[160]	TCTGAATGCGGTCTAATTTAATGCGGAGCGGGTCGTGCAGCTGAT	45

Supplementary Table 2. Sequences of the 3D DNA origami track

Start	End	Sequence	Length
Core staple strands			
10[48]	69[41]	TTATTTATCCCAATTGGGTTAAGAGTCATGCAACAGAACCTCAGTGTTTGAT TAAATTGCTTT	63
10[97]	27[97]	TCGTAAAATTGCCTGAATCCCAATGACC	28
11[35]	56[42]	AGCCTTTGAACACCAATCGTCGAATTACGTTGAAAGGAATTGCCTTGCT	49
11[84]	8[77]	TCAGGTCACTAGCAAAAAGCCTTTGTTAAAATTCG	35
12[62]	57[69]	CATTAGATTCCCTTTTACATTTTATCTAAAATATG	35
12[83]	45[90]	GGTAGCTTCTCACGGAAAAAGCCGTGGTGAAACAA	35
14[41]	52[28]	TATCTTAATTACCTAACGGATCAAACAATGAGTAACATTATC	42
14[62]	23[55]	AAACAATGAAATAGAGGAAACCCTCAGACGGAACC	35
15[21]	25[34]	GCCGAACCCTTTTTTATCAGAGAGATAATCTCTGATTGACAG	42
15[84]	55[90]	ATTTCAAGTAATGTGTAGGTAAAAAAATAAACGCGAGGCGCTTTCGCAC	49
16[122]	53[104]	TTTTTTATAAAGCTAAATCGGTTGTCAGCCTCCGGACTTACGGCAG	46
16[62]	53[62]	CCCAAAATAACGTCTCCTGATAGATGAT	28
17[28]	19[41]	CATAAAGGTGGCAAAATAGAAAATTCATCATTCAACCGATTG	42
17[70]	23[76]	CAGGCAATGCAGTAGCGACAGCCACCGGAACCGGA	35
19[91]	22[84]	GTCAATACGAACGAGTAGATTTGCAACTGCTGTAG	35
2[69]	35[55]	CTTGCCACTACGAAGGCACCAACAAAGTTTCGAGGAGGCTCC	42
2[76]	37[76]	AACCGAATATCATCGTATCGGTTTATCATAAAGGCCGCTTAA	42
21[56]	21[55]	TCGATAGAGGTGAATTATCACCGTCACCCACCAATGAAACCA	42
21[77]	21[76]	TTAAATATAGTTTGACCATATTCATTAACAGCACCGTAATTT	42
22[83]	19[83]	CTCAACAGGCAAAGAACATCCAATAAATTTTAGATACATTTC	42
23[35]	20[35]	ATAATCACAGACTGTAGCGCGAAGGCCGGAAACGTGACTTGA	42
23[77]	25[97]	GAGTACCTTTAATTCAACAGGGCCCGAAAGACTTC	35
23[98]	18[91]	TTGATAATTGCTGAAGCAATATCTACTAATAGTAG	35
25[35]	14[42]	GAGGTTGCCCTCAGCCAGAAGGAAACCGCAATAGC	35
25[42]	31[55]	AGGCAGGAATGGAAAAGTTTTTGCCTATGGGTTTTCGGAATAGCCACCACCC TCAT	56
25[56]	14[63]	ATTGGCCCCGCCACGCAATAATAACGTGAAGCAAG	35
25[77]	50[70]	AGAGGAATCAGGATAGCCTTTGACCCTGGTTTTTTTGGTGCTGGTCTTC	49
25[98]	15[122]	AAATATCCCGGAAGATAAAAATTTTTAGAACCCTTTTTT	39
26[104]	23[97]	CAGAAGCCACCATCAATGCAATGCCTGACGCAAGGCAAACTCGCTCCTT	49
27[56]	12[63]	TCAGTGCCTCATTACCAATAATAAGAAAAGAAGCG	35
27[77]	12[84]	GTTCAGACAAAAAGAAAGGCCGGAGACACGGAGAG	35
28[62]	45[62]	CAGTTAAATAAAAACAGGGAGACTACAAAATAAACAATGCTG	42
28[69]	11[83]	GTATAAAGGATAAGTGCCGGAGGGGGTATGCTTTAAAGGCTA	42
29[91]	46[98]	AATGTTTAGACTGGATTCATTGAGAGTCTGATAAAGAAACAGCCAGCAG	49
30[97]	35[97]	CTTTTGCAAAAGAAGAGCAACCAAAAGGACAGGTAAACGAACTATGCGATT TTAAG	56
31[56]	29[62]	TTTCAGGATATAAGTATAGCCGCTCAGTACCAGGC	35
31[77]	44[77]	ATAGTAAGTTTTGCTAATCAGTGTCAATCTCACCGGAAGGGA	42
33[35]	31[34]	AAAGTTTGTCACCAGTACAAACCGCCAC	28
33[56]	6[63]	ACGTTAGTGTACCGTCCCGACTTGCGTTGATTATT	35
33[77]	6[84]	CATTATTAATTACGTCAGCTCATTTTTTGCTTTCA	35
33[98]	31[97]	TCATCAGCAGATACATAACGCACTATCA	28

35[56]	3[69]	AAAAGGATTGCTAAAGCAAGCCGTTTGCCATCCTAAGATTTG	42
35[77]	4[77]	ATTACCTTAACGGAAACCCGTCGGATTCCATCTGCCAGTTTG	42
35[98]	33[97]	AACTGGCATCTACGTTAATAAGAAAGAT	28
36[90]	1[90]	CAACTTTAATCATTGCCTGATAAATTGTGTCAATCGGACAGA	42
37[35]	1[48]	TATTCGGCTTAAACATACCAAGCGCGAAACCTAAATTCCATT	42
37[56]	33[55]	AGGGAGTGCTTGCTACAACGGATTTACGAGCATGTGAGAACAACAACTTTTT CCAG	56
37[77]	2[77]	TCAACGTAACAAAGGGATATTCATTACCTGAAAGAATAAGGG	42
37[98]	38[98]	TTCAGTGAGATGGTTCCGCGAGCATCGTATCGCACCGGAAAC	42
39[21]	33[34]	AACGCGCCTGTTTAAATCGGCAAACCAAGGAGTGAACGATCT	42
4[48]	38[35]	AGAAACCAATCAATTCAACAATAAAGTA	28
4[76]	35[76]	AGGGGCCGAGTAACACCTGTATGGGATTGCCTTTAATTGTGA	42
4[97]	40[98]	AACCGTGTCCGTGGGAACAAACCTTCCTGGCGATCCGAAAGG	42
40[48]	38[56]	TGCGTTAAACATGTCAAAAGGTAGATAAGTCCTGAACAAGAAATATAAA	49
40[97]	10[98]	GGGATGTGTTTTCCTTAATATCCAAAAACAGGAAGACGGTAA	42
41[70]	33[76]	GAGCCCATAAATGTTGTTAAAAGAATAGGAACCCATAAATGAATTTTAA	49
42[34]	27[34]	TTTAGTTCTTAGGTCCAAATAAAATAGCCCTATTAAGTGTAC	42
42[90]	39[97]	GCCACGGCGCCAGGGCTGCAACAACTGTAGCGCCATCGGCCTCAGGAAG	49
42[97]	59[104]	TGGAGCCTCGGCGAAACGTACGAGATAGGCCTGTG	35
43[70]	31[76]	AATTAGTCATTAAACGGTTGACATCGAGAGGGTTGGATAGCAAGCCCGC	49
43[77]	60[84]	TGGGTAAGAACGGAGCGTGCCTGTTCTTTCGTAAT	35
44[62]	65[62]	GCTTAGAGCCAGCAGCAAAATTCAAACATCGCCATATATCCA	42
44[76]	28[70]	TAAACATAGCGATAATGCAAATCCAAACCATATGTATCTACAAATGCCC	49
46[34]	23[34]	CTTTTTTCATTTCACCGAAGCAAAGTTAAGCCACCTCTTTTC	42
46[97]	52[91]	TTGGGCGAAAAAGCCGCACAGGATGCTGGCCGGGCCAACCAGCTTACGG	49
47[42]	27[55]	GCTATTAGCTGAGATATAACTATATGTAAGCCATAGAATAACTGCCCCCAAC GGGG	56
47[70]	27[76]	TTGAAGCATTTTTGGGGTGAGATAACAAATAAATCCTTGAGTAACAGCA	49
47[91]	29[90]	AGACGCATTAATGCGTCAAATAAAGCGGATTGCATAAACGAGCCTCAAAAT AGTAA	56
49[56]	54[63]	GATGAAATTTTACATTAAATCCTTTGGGTTTCTTT	35
49[70]	25[76]	CAAGATAAAGATTCCGGGAGATACCTCCCTCAGAGTTGATATTCACATA	49
49[84]	54[77]	CCCGTAAGTTGTGTACATCGATAAACATTGGGTAA	35
50[55]	46[42]	TGTTTGGATTATACTAGATTTAAACAATGAGCAAATTCATTT	42
50[69]	16[70]	AATATAAAGATGAATATACCCTAATACT	28
51[49]	24[42]	TCAGGTTGAACTGGAAGAAACTTTGCCTTTAGCGTAAATCACACCGCCA	49
53[91]	48[84]	AATGCCAGTAGAACGTCAGCGCGTCTCGTTCCGGC	35
54[34]	16[21]	CCGTCAAGACTTTATCGCCTGATAAAGACTTATTACGCAGTA	42
54[62]	48[49]	AGGAGCACTAACAAACTCGTATCGGGAG	28
54[76]	49[69]	AGGTTTCTCATTGCGTAGTAACAGTACCCAAACAT	35
54[90]	65[83]	CTGGTAACCCTTACCCAGCGGCAGTGTCTTCACGGTCATACCGCTCACT	49
54[97]	16[84]	CCTGCGGTCAATCCATTGCCGTCGCTGGACCAAAAACATTAT	42
56[111]	69[104]	CAGATGCCGGGTTACACCAGTCTGGCCCCCTGTTT	35
56[69]	69[62]	GCTGAAAAATCTAAGCCACCGGCGGGAGACGTGCT	35
58[34]	47[34]	CAGCAGACACCGCCATAGTGATTCTGTA	28
58[48]	62[35]	ACCGAACCTTTAATTAGAACCCTTCTGACATTGGCAAATACC	42
59[42]	25[55]	GTGCCACGCTGAGATTAAGACATTAATTCGGGAGAGTTAAGCAAGCCAGTC AGACG	56

6[34]	42[35]	ATAGCAACTAAGAACGCGAGGTTAGTTGATAAGGCAATATAT	42
6[62]	9[62]	TTCATCGGCAGAGGTGTTTAGTATCATAAAGAATAGCCTTAACAGAGCCTAA TTTG	56
6[83]	41[69]	TCAACATGGCTGCGGGCGATTAACTAGAAAAAGCCCATTTTC	42
60[69]	58[49]	GGACATTCTGGCCACACGACCCAACAGGAAAAACGGCTGGTATAAAAAT	49
60[83]	43[69]	CATGGTCATAGCAGCCAGCACTATCGCAAGACAAAGAATCAT	42
62[104]	44[91]	ATGAGTGAGCTAACTTCCAGTGGCCGTTACTGCGCACTTTCT	42
62[34]	71[41]	TACATTTAGAACTCAAACTATCTTCTTTGGAAGAA	35
64[62]	49[55]	AAATTAACCGTTGTCAGTGAGAGCATCAAGGAAGGTAACAATAGAAGAT	49
64[83]	69[83]	TGCGTATACTATTATTGTTCCAGTTTGGTTGCTGGTTTGCCC	42
65[70]	56[77]	ATTACGCGGGGGTTCAGACGATCCAGCGTGCCGGT	35
65[98]	69[97]	CGGGAAAAACGCGCCAACGTCAGCCCGAGATAGGGCGAAAAT	42
66[27]	56[22]	TAGACAGCCAGAATCCTGAGAAAATATCAAACCC	34
66[48]	63[48]	GGAGGCCTTATAATAGCAATACGGCCTTCTCATGGAGATTCA	42
66[97]	63[90]	TGAGAGAGTTGCAGGTGGTTTGGCGGTTGCCCGCTTCACATTGAAATTG	49
67[70]	47[69]	GAGTCGGGCCCCCTCTCGTCACAAAACAAAATTAAAGAATCC	42
67[77]	67[69]	CGCCAGGCAAGCGGTCCACAGAATCAGAAGTAAAA	35
68[41]	66[28]	GTAACCACCACACCACTATGGGGGATTT	28
7[49]	6[35]	GCGAACCTAACACTGAGTTTCTGTCGTCTCAACAGTTTCAGCGTACCGCGCG CCCA	56
9[28]	28[35]	CAACGCTCACCGTAGATTAGGATTAGCGTTCGGAA	35
9[63]	61[69]	CCAGTCCTTGGAGGTTTTGAAAACACCGGAACGCGCTGATAGCCCTATG	49
Staple strands in foothold row A			
1[28]	35[34]	TCTGAATGCGAACTGGTTTTCATGAGGAAGTACGAAAGAGGCAAAAGCGAT TAGCTTGACGTTGAA	66
1[49]	37[34]	TCTGAATGCGAACTGGTAAACGGGAGCAGCGAAAGACAGCATCGGAACGA GGACCGATA	59
1[70]	1[69]	TCTGAATGCGAACTGGTCCAACTTCATTGCGGGATCGTCACCCTCTAAAATA CGTAAGA	59
1[91]	36[91]	TCTGAATGCGAACTGGTTGAACGGTGTACAGACGAGGCGCAGACGGTCGAA ATTAATTT	59
1[28]	35[34]	TCTGAATGCGAACTGGTTTTCATGAGGAAGTACGAAAGAGGCAAAAGCGAT TAGCTTGACGTTGAA	66
Staple strands in foothold row B			
38[34]	43[34]	TCTGAATGCATGCAAGGATTCTGTTATTTAACAACGCCTACAAATTCTTACC GTGATAA	38[34]
38[55]	5[48]	TCTGAATGCATGCAAGGGTACCGAAATTTAGTAGGAATCATTACCACTCATC	38[55]
38[76]	38[77]	TCTGAATGCATGCAAGGTTAGTAATAAGAGAAAATAATATACGACGACAGT ATTCGCCA	38[76]
38[97]	8[98]	TCTGAATGCATGCAAGGCAGGCAATGGGAAGGTAGCCAAACCAATAGGAAC GGTAAACG	38[97]
38[34]	43[34]	TCTGAATGCATGCAAGGATTCTGTTATTTAACAACGCCTACAAATTCTTACC GTGATAA	38[34]
Staple strands in foothold row C			
63[28]	58[35]	TCTGAATGCTGGATCTCTTATTTACCTGAAAGAATGGCTATTAGTGAACCAC	52
63[49]	43[48]	TCTGAATGCTGGATCTCCCAGTCAACAGAGAGCGCGAAAGAAAACTTTTTC AGTTAAAT	59
63[70]	63[69]	TCTGAATGCTGGATCTCAATGTTTCCTGTGTAATTGCGTTCGCCAGCCATTGA GTAATA	59
63[91]	58[98]	TCTGAATGCTGGATCTCTTATCCGCTCGAATCGCGTCCGTGAGCCTGCGGCG	52
63[28]	58[35]	TCTGAATGCTGGATCTCTTATTTACCTGAAAGAATGGCTATTAGTGAACCAC	52
Staple strands in foothold row D			
70[34]	64[21]	TCTGAATGCGACTCTAATTTAGAGAAGGAAGGATTAGTAATAACA	45
70[55]	70[56]	TCTGAATGCGACTCTAAAACCCTAGGAGCGGGCGCTAGGGCGCCAAAGCAC TAAATCGG	59

70[76]	65[69]	TCTGAATGCGACTCTAATGCCGTAAGAGTCCTGTGTCCATCACGCGAACAAT	52
70[97]	57[104]	TCTGAATGCGACTCTAATCAAGTTTGGACTCGGGGAGATTCTTTTCACCAGT	52
70[34]	64[21]	TCTGAATGCGACTCTAATTTAGAGAAGGAAGGATTAGTAATAACA	45
Staple strands in foothold row E			
69[105]	70[98]	TCTGAATGCGGAGAGTTGATGGTGAAAGAATAAAGGGCAACCATCACCCAAA	52
69[42]	70[35]	TCTGAATGCGGAGAGTTGACGAGCGCTGCGCAGCGAAAAAGGGAGCCCCCGA	52
69[63]	66[49]	TCTGAATGCGGAGAGTTTTCCTCGAATGGCAAGTGTAGCGGTCACACGTATACTAAACA	59
69[84]	70[77]	TCTGAATGCGGAGAGTTCAGCAGGTTGAGTGAAGAACGTTTTGGGGTCGAGG	52
69[105]	70[98]	TCTGAATGCGGAGAGTTGATGGTGAAAGAATAAAGGGCAACCATCACCCAAA	52
Staple strands in foothold row F			
53[105]	54[98]	TCTGAATGCGGTCTAATCACCGTCTCCCACGGCGGTTGCGGGTCACTGTTGC	52
53[42]	54[35]	TCTGAATGCGGTCTAATTATTCCTAAAAGTTTTCGACACTAATAGATTAGAG	52
53[63]	53[55]	TCTGAATGCGGTCTAATCCGCAAGCTGGAGGTGTCCAGCATCAGCCAGGTCAGCAGCAA	59
53[84]	53[83]	TCTGAATGCGGTCTAATGGCAATTGGCCCGAACGTTATTAATTTTGATTATC	52
53[105]	54[98]	TCTGAATGCGGTCTAATCACCGTCTCCCACGGCGGTTGCGGGTCACTGTTGC	52
Staple strands in foothold row G			
19[105]	19[104]	TCTGAATGCTAGAAGTCTAGCTATATTTTCAAACAGTTGATTCCCAATTCTGACCTGTT	59
19[42]	53[41]	TCTGAATGCTAGAAGTCAGGGAGGCACAATCCATATAACATGATTAAGACTC	52
19[63]	19[62]	TCTGAATGCTAGAAGTCACGGAAACACGGAATAAGTTTATTTTGTGAAGGTAAATATTG	59
19[84]	21[97]	TCTGAATGCTAGAAGTCGCAAATGTAGCATTAATTAGCAAAATTAATATAATAAAGTAC	59
Staple strands for stators			
23[14]	26[21]	ACAACAACAATTAGCGTTTGCCAACCCTCAGCCAGCAATTTACC	44
23[56]	16[63]	ACAACAACAAGAGCCAAATCAAGGCAAAGACACCATATTGAATA	44
27[14]	30[21]	ACAACAACATTTGATGATACAGGTTCTGAAAGAGAAGCTCAGGA	44
27[35]	11[48]	ACAACAACATGGTAATAGCGCAGCCCACAAGAATTGAATTAACTACAGAGA	51
31[14]	34[21]	ACAACAACACGCCACCCTCAGAACTACAACTAGCGTAGAATAGA	44
31[35]	7[48]	ACAACAACACCTCAGAGGTGTATAACGAGCGTCTTTCATCAAGACGTTTTA	51
35[14]	37[27]	ACAACAACATAATAATTTTTTCATACCGATACGCATA	37
35[35]	37[55]	ACAACAACAAATCTCCAAAAAAATGAATTTTCGCTGAGGCTTGC	44

Supplementary Table 3. Sequences of strands on AuNRs, blocking strands and removal strands

Strand	Sequences
Strands on walker	GCA TTC AGA TTTT (3’-SH)
Strands on stator	TGT TGT TGT TTTT (3’-SH)
Blocking strand a	ACCAGTTCGCATTCTCTAGCTTACT
Removal strand $\bar{a}$	AGTAAGCTAGAGAATGCGAACTGGT
Blocking strand b	CCTTGCATGCATTCTCTCCTTATCG
Removal strand $\bar{b}$	CGATAAGGAGAGAATGCATGCAAGG
Blocking strand c	GAGATCCAGCATTCTTTGTACGAAC
Removal strand $\bar{c}$	GTTCGTACAAAGAATGCTGGATCTC
Blocking strand d	TTAGAGTCGCATTCTATTAGCAACG
Removal strand $\bar{d}$	CGTTGCTAATAGAATGCGACTCTAA
Blocking strand e	AACTCTCCGCATTCTCTCACTAATT
Removal strand $\bar{e}$	AATTAGTGAGAGAATGCGGAGAGTT
Blocking strand f	ATTAGACCGCATTCTTTGAGTTCCG
Removal strand $\bar{f}$	CGGAACTCAAAGAATGCGGTCTAAT
Blocking strand g	GACTTCTAGCATTCCTTCACTTTCA
Removal strand $\bar{g}$	TGAAAGTGAAGGAATGCTAGAAGTC

Supplementary Table 4. Addition procedures for the CD spectrum measurements

	first addition	second addition	third addition	fourth addition
I	1 µL H_2O	1 µL H_2O	1.5 µL H_2O	1.5 µL H_2O
II	0.5 µL a, 0.5 µL $\bar{c}$	1 µL H_2O	1.5 µL H_2O	1.5 µL H_2O
III	0.5 µL a, 0.5 µL $\bar{c}$	0.5 µL b, 0.5 µL $\bar{d}$	1.5 µL H_2O	1.5 µL H_2O
IV	0.5 µL a, 0.5 µL $\bar{c}$	0.5 µL b, 0.5 µL $\bar{d}$	1 µL c, 0.5 µL $\bar{e}$	1.5 µL H_2O
V	0.5 µL a, 0.5 µL $\bar{c}$	0.5 µL b, 0.5 µL $\bar{d}$	1 µL c, 0.5 µL $\bar{e}$	1 µL d, 0.5 µL $\bar{f}$

Supplementary Table 5. Samples added to drive the plasmonic walker for the real-time CD detection

Step	strands added
I → II	0.5 µL a, 0.5 µL $\bar{c}$
II → III	0.5 µL b, 0.5 µL $\bar{d}$
III → IV	1 µL c, 0.5 µL $\bar{e}$
IV → V	1 µL d, 0.5 µL $\bar{f}$
V → IV	1 µL f, 1 µL $\bar{d}$
IV → III	1 µL e, 1 µL $\bar{c}$
III → IV	1.5 µL c, 1.5 µL $\bar{e}$

Supplementary Note 1: Walking mechanism

The plasmonic walker is driven by addition of the blocking and removal strands, gaining energy from DNA hybridization. In contrast to traditional DNA walkers, first, our walking element is an anisotropic AuNR with a dimension of 35 nm×10 nm. The walker AuNR should be perpendicular to the stator AuNR during walking. Second, it is difficult to obtain stable AuNRs with discrete numbers of foot strands at similar buffer conditions as those of the DNA walkers. Therefore, the feet, the track, and the fuel strands (including the blocking and removal strands) had to be carefully designed. Different from conventional bipedal DNA walkers that have two feet (normally with different sequences) and walk in a bipedalism manner, the plasmonic walker is covered by multiple feet (estimated to be several hundreds) with identical DNA sequences and walks in a "rolling" fashion.

Six rows of foothold strands are extended from the DNA origami track. Each row of the footholds consists of five identical foothold strands. Each foothold strand contains two segments: one 9-nt binding segment fully complementary to the foot strand that is used to bind the walker and the other segment is an 8-nt toehold. The toehold segment is directly extended from the origami to ensure it is still accessible when the walker AuNR attaches to the binding segment of the foothold. Due to the fact that the binding segments are identical for all of the footholds, different rows of the footholds are distinguished by their toehold segments which are specifically sequenced for different rows. Blocking strands are used to deactivate their corresponding footholds by hybridization through a toehold-mediated strand-displacement reaction. Each blocking strand contains three segments. One is the 8-nt segment to hybridize with the toehold on foothold; the middle segment is 6-nt, which can hybridize with the binding segment on the toehold; the rest segment is an 11-nt toehold which enables the removal of the blocking strand from its foothold by a removal strand, which is fully complementary with the blocking strand. The blocking strand can hybridize with its foothold, forming in total 14-bp hybridization, stronger than that formed by the foot strand and foothold (9-bp). This ensures that the foot strand can be released from the foothold. The existence of the 3-nt non-complementary difference between the blocking strand and the binding segment on the foothold makes the blocking strand only have a 6-nt complementary part with other footholds, therefore unlikely to be stable at room temperature in this buffer condition. More importantly, this avoids undesirable binding between the removal strands and foot strands on the AuNRs.
